# From Proteostasis Collapse to Endothelial Failure: Mechanistic Pathways Linking Heat Stress, Regulated Cell Death, and Coagulopathy

**DOI:** 10.3390/ijms27219804

**Published:** 2026-10-31

**Authors:** Emina Dervišević, Temima Ajanović, Nina Čamdžić, Monica Salerno, Cristoforo Pomara, Massimiliano Esposito, Mario Chisari, Amina Zorlak, Nejra Selak, Enisa Hodžić, Francesco Sessa

**Affiliations:** 1 Department of Forensic Medicine, Faculty of Medicine, University of Sarajevo, 71000 Sarajevo, Bosnia and Herzegovina; emina.dervisevic@mf.unsa.ba (E.D.); amina.zorlak@mf.unsa.ba (A.Z.); 2 Department of Chemistry, Faculty of Science, University of Sarajevo, 71000 Sarajevo, Bosnia and Herzegovina; temima.ajanovic@gmail.com; 3 Department of Pathology, Faculty of Medicine, University of Sarajevo, 71000 Sarajevo, Bosnia and Herzegovina; nina.camdzic@mf.unsa.ba (N.Č.); selaknejra@gmail.com (N.S.); 4 Department of Medical, Surgical and Advanced Technologies “G.F. Ingrassia”, University of Catania, 95121 Catania, Italy; monica.salerno@unict.it (M.S.); cristoforo.pomara@unict.it (C.P.); 5 Faculty of Medicine and Surgery, “Kore” University of Enna, 94100 Enna, Italy; massimiliano.esposito@unikore.it (M.E.); mario.chisari@unikore.it (M.C.); 6 Department of Internal Medicine and Cardiology, Clinic for Heart, Blood Vessel and Rheumatic Diseases, Sarajevo University Clinical Centre, 71000 Sarajevo, Bosnia and Herzegovina; enisa.hodzic@mf.unsa.ba; 7 Department of Psychology and Health Sciences, Faculty of Human Sciences, Education, and Sports, Pegaso University, 80143 Naples, Italy

**Keywords:** heatstroke, hyperthermia, proteostasis collapse, regulated cell death, apoptosis, heatstroke-induced coagulopathy, oxidative stress

## Abstract

This review proposes an integrative mechanistic model that brings together apoptosis, pyroptosis, ferroptosis, necroptosis, and necrosis within the broader context of heat-induced proteostasis disruption. Rather than depicting a strictly sequential or fully validated causal pathway, the model provides a conceptual framework through which interactions among cellular stress responses, endothelial dysfunction, heatstroke-induced coagulopathy (HIC), and multiple organ dysfunction syndrome (MODS) may be understood. Under extreme heat stress, widespread protein unfolding and chaperone saturation activate the unfolded protein response, disrupt endoplasmic (ER) calcium homeostasis, and couple through Ca^2+^-mediated injury to mitochondrial dysfunction and oxidative stress. These processes are reciprocally amplifying: reactive oxygen species (ROS) from failing mitochondria deepen proteostatic damage and potentiate necroptotic and ferroptotic signaling, while inflammation-driven immune activation further increases ROS production, closing a self-reinforcing cycle. The resulting engagement of multiple RCD pathways, apoptosis, pyroptosis, ferroptosis, and necroptosis, each with qualitatively distinct DAMP signatures, promotes inflammasome activation and a cytokine storm that destabilizes the endothelial glycocalyx, impairs barrier integrity, and triggers a pro-coagulant shift underlying HIC. We further examine biomarker candidates reflecting these mechanisms, evaluate emerging therapeutic strategies, ranging from HSP modulation and antioxidant therapy to endothelial-protective agents, mitophagy regulators, and nanomedicine, and outline the future role of multi-omics, machine learning, and regenerative approaches. By bringing together molecular, cellular, vascular, and systemic processes into a single cohesive model, this review proposes a conceptual foundation to guide diagnostic refinement, therapeutic innovation, and forensic interpretation in severe heat-related injury.

## 1. Introduction

Heat stress and heat-related illnesses represent an expanding global health challenge as climate change, urban heat islands, and extreme thermal exposures become increasingly common. In recent years, surface temperatures as high as 40–46 °C have been reported across multiple countries, placing vulnerable populations, outdoor workers, and athletes at significant risk of heat-induced systemic injury [1]. Heatstroke, a life-threatening condition resulting from the failure of thermoregulatory mechanisms, is characterized by severe hyperthermia, systemic inflammation, endothelial dysfunction, coagulopathy, and multiorgan failure. Mortality can be substantial: for example, a Japanese survey reported a 4.6% fatality rate among hospitalized heat-illness patients [2]. Furthermore, the International Laboure Organization estimates that 153 workers worldwide sustain work-related injuries every 15 s, with thermal injury among the most severe categories [3]. These epidemiological observations underscore the urgent need for deeper mechanistic understanding and more effective clinical strategies to predict, detect, and mitigate heat-induced organ damage.

Despite progress in characterizing classical heatstroke physiology, important knowledge gaps remain. Although several reviews have examined specific components of heatstroke pathophysiology, including inflammation, endothelial dysfunction, coagulopathy, mitochondrial damage, oxidative stress, and regulated cell death, these processes are typically considered separately. To our knowledge, no previous review has integrated these mechanisms within a proteostasis-centered framework linking intracellular thermal injury to systemic disease progression and multiorgan failure. This unresolved gap forms the basis of the present review. Current models insufficiently integrate how molecular injury processes, such as proteostasis collapse, oxidative stress, and regulated cell death (RCD), combine to drive systemic inflammation, endothelial barrier failure, disseminated intravascular coagulation (DIC), and multiorgan dysfunction syndrome (MODS) [1,2,3,4,5]. Mechanistic explanations describing the transition from localized cellular damage to organ-level failure remain fragmented across disciplines. For example, while extensive research has examined heat shock proteins (HSPs), endoplasmic reticulum (ER) stress, mitochondrial dysfunction, or individual cell death pathways, these processes are usually discussed in isolation, lacking a unifying pathogenic sequence. Additionally, the interdependence of Damage-Associated Molecular Pattern (DAMP) release, innate immune activation, endothelial glycocalyx degradation, and microvascular thrombosis has not been systematically articulated, even though these mechanisms are central to heatstroke-induced coagulopathy (HIC) and its clinical progression [2,6]. This fragmentation limits the ability of clinicians and researchers to predict deteriorating patients, develop targeted therapies, or interpret biomarker signatures in thermal injury.

Current literature describes important components of heat injury, HSP biology, ER stress, mitochondrial dysfunction, pyroptosis, ferroptosis, glycocalyx shedding, microthrombosis, but these remain fragmented across disciplines. Within the broader field of inflammatory and immunogenic cell death, these processes are increasingly recognized as central mediators of sterile inflammation and tissue injury across multiple pathological conditions, including sepsis, trauma, and ischemia–reperfusion injury.

An important terminological clarification is warranted at the outset. The conditions encompassed by thermal pathology differ substantially in their etiology, injury pattern, and systemic progression, and should not be treated as interchangeable. Heatstroke is a systemic illness defined by a core body temperature exceeding 40 °C accompanied by central nervous system dysfunction (delirium, seizures, or coma), resulting from failure of the body’s thermoregulatory mechanisms [1]. It is classified as classic (passive environmental exposure, typically affecting elderly or immunocompromised individuals) or exertional (occurring during strenuous physical activity in young, active persons) [2,3,4].

In heatstroke, the primary insult is a whole-body rise in core temperature that triggers systemic proteostasis collapse, gut barrier failure with endotoxaemia, a generalized inflammatory response, endothelial dysfunction, coagulopathy, and ultimately multiorgan failure. Heat stress and hyperthermia denote the earlier, reversible stages of a heat-illness continuum that ranges from mild conditions (heat edema, heat syncope, heat cramps, heat exhaustion) to heatstroke as its most severe endpoint; progression occurs when compensatory thermoregulatory mechanisms, increased cardiac output, cutaneous vasodilation, and sweating, are overwhelmed by dehydration and circulatory failure, causing the hypothalamic thermoregulatory center to lose control and core temperature to rise rapidly beyond 40 °C [5]. In contrast, burn injury (thermal trauma) is a localized insult caused by direct contact with a heat source (flame, hot liquid, hot surface, or radiation), producing concentric zones of coagulation necrosis, stasis, and hyperaemia at the wound site [6]. When burns are extensive (typically ≥20–30% total body surface area), the wound-driven inflammatory response can escalate into a systemic syndrome (SIRS) that shares downstream mediators (DAMPs, cytokine storm, endothelial activation, DIC) with heatstroke but originates from a fundamentally different initiating event: massive tissue destruction rather than thermoregulatory failure [7]. Smoke inhalation injury represents yet another distinct entity, involving chemical and particulate damage to the airway epithelium, surfactant dysfunction, and systemic toxicity from carbon monoxide and hydrogen cyanide, which may coexist with cutaneous burns but follows its own pathophysiological trajectory [8,9]. Because the mechanistic framework proposed in this review explores proteostasis collapse as a potential shared upstream contributor to regulated cell death and subsequent endothelial–coagulopathic injury, we draw evidence from both heatstroke-specific and burn-derived studies where the molecular pathways converge. A substantial proportion of the mechanistic evidence discussed in this section originates from experimental and clinical studies of severe burn injury rather than heatstroke itself. While these conditions share several pathophysiological features, including thermal stress, systemic inflammation, endothelial dysfunction, coagulopathy, microvascular injury, and multiorgan failure, important differences exist regarding injury mechanisms, exposure characteristics, and clinical evolution. Consequently, findings derived from burn models should be interpreted cautiously when extrapolated to heatstroke. In the present review, burn-related evidence is used primarily to provide mechanistic insights and to identify biologically plausible pathways that may also contribute to organ dysfunction in heatstroke, particularly where direct heatstroke-specific data remains limited.

Current literature describes important components of heat injury, HSP biology, ER stress, mitochondrial dysfunction, pyroptosis, ferroptosis, glycocalyx shedding, microthrombosis, but these remain fragmented across disciplines. However, accumulating evidence from both heat stress and burn injury models suggests that these processes may be mechanistically interdependent. Importantly, evidence supporting these relationships originates from different experimental settings, and the extent to which observations from burn injury can be extrapolated to heatstroke remains incompletely established [8,9]. When heat stress is strong or prolonged, the HSF1-driven heat shock response is overwhelmed, causing proteostasis collapse, accumulation of misfolded proteins, and activation of further stress pathways [10]. In hyperthermia, intense or prolonged heat saturates proteasomal capacity, leading to formation of insoluble protein inclusions that directly contribute to cell death, mitochondrial dysfunction, and the release of intracellular components including mtDNA [11]. Within the ER, thermal denaturation of proteins activates the unfolded protein response (UPR) through the PERK, ATF6, and IRE1/XBP1 branches; in heat-stressed intestinal epithelial cell models, moderate UPR activation reduces cell stress and inflammation by blocking NF-κB, but excessive or prolonged ER stress triggers apoptotic signals through CHOP, consistent with elevated GRP78/BiP and CHOP levels observed in the context of heatstroke-associated protein denaturation [12]. UPR failure further disrupts calcium homeostasis and increases ROS production [12]. In heat-exposure models, heat stress downregulates SERCA activity and precipitates intracellular Ca^2+^ overload, linking Ca^2+^ dysregulation directly to ER stress-mediated injury [13,14]. Critically, Ca^2+^ overload couples ER stress to mitochondrial injury: in endothelial models, intense heat stress induces apoptosis through a Ca^2+^-mediated mitochondrial pathway involving mitochondrial Ca^2+^ uptake and loss of mitochondrial integrity [15], while in vivo evidence confirms that heat-induced Ca^2+^ leakage drives mitochondrial damage and tissue breakdown [16]. In heat stress, one major consequence of losing proteostasis is the generation of ROS and oxidative stress, which directly damages mitochondria, causing loss of mitochondrial membrane potential, release of cytochrome c, and activation of the intrinsic apoptotic pathway [17]. In burn injury patients, mitochondria have been identified as one of the main sources of ROS after thermal trauma; damage to the mitochondrial membrane leads to reduced respiratory chain activity, increased superoxide production, and impaired ATP synthesis [18], while clinical and experimental data from both burn and heatstroke settings confirm that inflammation and oxidative stress mutually reinforce each other in a vicious cycle [19,20]. ROS also potentiate necroptotic signaling in heat-exposed intestinal epithelial cell models, high temperatures trigger necroptosis through the RIP1–RIP3–MLKL pathway, and treatment with the antioxidant N-acetyl-L-cysteine attenuates RIP1/RIP3-mediated cell death [21]. Released mtDNA activates TLR9, the cGAS–STING pathway, and the NLRP3 inflammasome, amplifying inflammatory signaling, cytokine production, and endothelial activation [22], and leading to increased production of IL-1β and IL-18 [23]. In heat-exposed intestinal cells, ferroptosis, involving iron-dependent lipid damage and GPX4 reduction, and PANoptosis, a convergence of pyroptosis, apoptosis, and necroptosis, together amplify inflammation and barrier [24]. Denatured and oxidatively modified proteins, including carbonylated proteins and AOPP, act as DAMPs recognized by TLR4 on innate immune cells, engaging NF-κB and inflammasome pathways and sustaining production of TNF-α, IL-1β, IL-6, and MCP-1; this DAMP-mediated signaling has been shown to contribute to sterile systemic inflammation characteristic of severe thermal injury [25]. In burn patients, activation of the NF-κB pathway and cytokine release further enhances ROS production through activation of neutrophils and macrophages [19], a mechanism also documented in heatstroke closing the self-amplifying loop [20]. Together, as demonstrated across heat-stress and burn-injury models, ion channel dysregulation, Ca^2+^ overload, and ER stress form a tightly interconnected network that amplifies cellular injury during severe heat exposure [13], linking membrane-level perturbations to downstream pathways of mitochondrial dysfunction, inflammatory activation, and cell death. It is this network of reciprocal amplification, rather than any single pathway in isolation, that this review seeks to formalize into a unified mechanistic model.

By integrating insights that span the molecular, cellular, vascular, immunological, and systemic levels, this review offers a comprehensive framework for understanding how heat exposure progresses from an initial intracellular insult to full-blown multisystem failure. Rather than treating these processes as isolated phenomena, it traces a continuous pathological sequence: thermal stress disrupts proteostasis within the cell, triggering regulated forms of cell death; these dying cells release DAMPs that amplify inflammation; the ensuing immunological pressure destabilizes the endothelium, which in turn precipitates coagulopathy; and together these events culminate in MODS. Presenting these interconnected mechanisms as a unified whole not only deepens the mechanistic understanding of heat-induced injury but also creates a clearer foundation for identifying meaningful biomarkers, prioritizing therapeutic targets, and refining diagnostic tools. This integrated perspective is essential for improving clinical management and strengthening forensic evaluation in cases of severe or fatal heat exposure.

While the primary focus of this study is heatstroke and severe heat-induced systemic injury, information from burn injury, thermal trauma, and systemic inflammatory models also informs key mechanistic concepts presented throughout the text. There are differences between these diseased conditions. Systemic hyperthermia, thermoregulatory failure, endothelial dysfunction, gut-derived endotoxemia, and multiorgan injury are the main characteristics of classic and exertional heatstroke, while burn injury also includes localized tissue necrosis, wound-associated inflammation, and, in certain situations, smoke inhalation injury. Proteostasis disruption, oxidative stress, regulated cell death, DAMP-mediated inflammation, endothelial activation, microvascular dysfunction, and coagulopathy are among the shared pathophysiological mechanisms that these medical conditions share. In order to foster a more comprehensive conceptual understanding, certain mechanistic results from burn damage and related thermal stress models were used where direct heatstroke-specific information was limited.

In summary, this review proposes a new integrative model in which proteostasis collapse may represent an initiating mechanistic event linking cellular thermal injury to systemic endothelial dysfunction and coagulopathy. This integration is achieved by systematically connecting upstream proteostasis collapse with downstream mitochondrial dysfunction, ROS amplification, regulated cell death pathways, and vascular–coagulopathic outcomes within a single continuous mechanistic framework, rather than treating these processes as independent events. The idea that proteostasis breakdown may act as a key mechanistic interface linking many pathogenic processes in heatstroke is examined in this review. Importantly, although individual components of this pathogenic cascade are supported by experimental evidence, including heat-induced protein denaturation, ER stress, mitochondrial dysfunction, oxidative stress, regulated cell death, endothelial activation, and coagulopathy, direct evidence demonstrating that proteostasis collapse functions as the single upstream driver coordinating all of these processes remains limited. Future studies specifically designed in heatstroke models will be necessary to validate the proposed mechanistic links. The search strategy has been described in Supplementary Materials.

## 2. Heat-Induced Proteostasis Collapse

Heat exposure threatens the stability of the proteome by altering the folding state, solubility, and interactions of proteins across nearly all cellular compartments. Because most proteins evolved to function within a narrow physiological temperature range, even mild hyperthermia can induce protein unfolding, membrane fluidization, and cytoskeletal destabilization, prompting cells to activate conserved protective mechanisms collectively known as the cellular stress response (CSR) [26,27]. Central to this response is the heat shock response (HSR), orchestrated primarily by heat shock factors (HSFs) and mediated through the rapid induction of molecular chaperones, particularly members of the HSP100, HSP90, HSP70, HSP60, and small HSP families [27].

### 2.1. Heat Shock Response, Chaperones, and Thermotolerance Thresholds

In unstressed cells, the transcription factor HSF1 exists in an inactive monomeric form bound to chaperones such as HSP70, HSP90, and HSP40. Thermal stress increases the pool of unfolded or partially denatured proteins, which compete for chaperone binding and release HSF1, allowing it to trimerize, translocate to the nucleus, and bind heat shock elements (HSEs) to initiate HSP transcription [25,26]. Once translated, HSPs promote refolding of misfolded proteins, disaggregate protein clusters, stabilize cytoskeletal components, and guide irreversibly damaged proteins toward degradation. This coordinated program forms the biochemical basis of thermotolerance, enabling cells to temporarily withstand elevated temperatures.

Importantly, heat stress produces graded effects depending on temperature and exposure duration. Temperatures of 39–41 °C typically activate the HSR without causing irreversible injury. At more severe thresholds (e.g., ≥43–44 °C), protein unfolding becomes widespread, proteasomal workload increases dramatically, and protein aggregation accelerates [28]. Experimental models show that exposure to 44–46 °C causes rapid accumulation of protein aggregates, cytoskeletal collapse, and early reductions in cell proliferation, even when HSP expression is robust. These findings highlight that the HSR has a finite buffering capacity and that proteostasis collapse is a threshold-dependent event driven by the kinetics of protein unfolding and the saturation of chaperone systems.

### 2.2. From Adaptive Protection to Proteostasis Failure

Persistent or intense hyperthermia gradually pushes the cell beyond the protective capacity of the heat shock response [26]. What begins as an adaptive reaction soon becomes unsustainable as the continuous influx of misfolded proteins overwhelms the available chaperone networks. Once HSP70, HSP90, and the small heat shock proteins are fully occupied, they can no longer stabilize essential enzymes, cytoskeletal elements, or mitochondrial proteins. As a result, key metabolic and structural proteins lose their function, organelles begin to lose their integrity, and misfolded proteins start to accumulate in large aggregates that the cell can no longer manage. At the same time, the ubiquitin–proteasome system becomes saturated by the rising burden of damaged proteins. Because the proteasome cannot clear ubiquitinated substrates fast enough, these proteins aggregate into insoluble inclusions that further interfere with normal cellular processes [26].

In parallel, the mitochondria, already strained by the increased demand for energy, begin to malfunction. Their chaperone-mediated import and quality control systems fail, destabilizing the respiratory chain and triggering a surge in ROS. These reactive species, in turn, amplify cellular injury by oxidizing lipids, proteins, and DNA, creating a vicious cycle of progressive proteostasis collapse [29].

As these stressors accumulate, the cell can no longer rely on adaptive pathways and instead shifts toward various forms of RCD. Depending on the intracellular conditions, this may involve intrinsic apoptosis driven by cytochrome c release and caspase activation, ferroptosis resulting from iron-dependent lipid peroxidation, necroptosis mediated by RIPK1/RIPK3/MLKL signaling, or pyroptosis triggered by gasdermin-dependent membrane rupture. The simultaneous activation of these overlapping death pathways reflects the extent to which both proteostatic and metabolic homeostasis have been lost [11,12].

**Figure 1:**
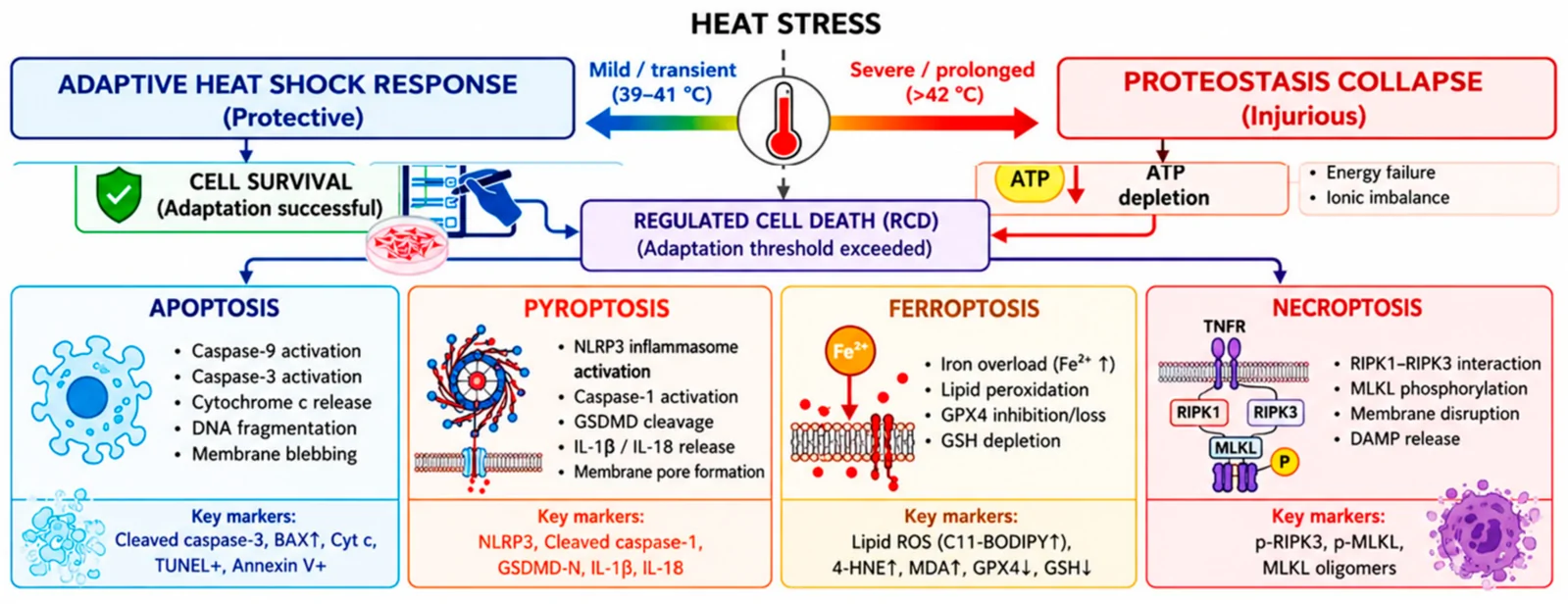
Under thermal stress, the mechanism shifts from adaptive heat shock responses to proteostasis collapse and controlled cell death. Through HSF1 activation, HSP70/HSP90 induction, and improved protein refolding, mild heat exposure triggers the adaptive heat shock response, preserving cellular proteostasis and survival. Proteostatic capacity is surpassed when heat stress intensifies or persists, leading to protein aggregation, proteasomal overload, endoplasmic reticulum stress, oxidative stress, mitochondrial dysfunction, and ATP depletion. Cells move toward controlled cell death pathways, such as apoptosis, pyroptosis, ferroptosis, and necroptosis, once this adaptive threshold is exceeded. DAMP release, inflammation, endothelial damage, and the emergence of organ dysfunction linked to heat stress are all caused by these mechanisms. Created in BioRender. Ajanovic, T. (2026); https://BioRender.com/4ghfdnu.

Eventually, when chaperone reserves are exhausted and mitochondrial ATP production collapses, the cell loses the ability to maintain ion gradients, membrane integrity, and redox balance. At this stage, injury becomes irreversible. The cell releases large quantities of DAMPs, fueling inflammatory signaling and propagating damage to surrounding tissues. What began as an attempt to restore homeostasis thus transitions into a self-amplifying process of cytotoxicity, inflammation, and tissue-level dysfunction (Figure 1).

## 3. Interplay of Regulated Cell Death Pathways and Progression to Accidental Cell Death

Cell death can be broadly categorized as accidental cell death (ACD) or regulated cell death (RCD), a distinction that is fundamental to understanding the pathophysiology of heatstroke [27].

Following the NCCD 2018 recommendations, each RCD modality is characterized according to its essential molecular machinery, the minimum set of molecular events that are both necessary and sufficient for that form of cell death, rather than by morphological appearance alone. This is important because morphologically similar outcomes (e.g., cell swelling and membrane rupture) can result from molecularly distinct programs (necroptosis vs. pyroptosis vs. ferroptosis) with different upstream triggers, different inhibitor sensitivities, and critically, different immunological consequences [27].

An additional distinction that is relevant to the present review is between physiological and pathological RCD. Under homeostatic conditions, apoptosis serves essential physiological functions: developmental morphogenesis, lymphocyte selection, and epithelial turnover in the intestine [27]. These processes are immunologically silent because apoptotic cells are rapidly cleared by phagocytes before membrane integrity is lost. In heatstroke, the same RCD programs become pathological when they are activated simultaneously in multiple tissues at a scale that overwhelms normal clearance and repair capacity.

A critical organizing principle for this review is the distinction between non-lytic and lytic RCD, which determines the immunological consequences of cell death and the nature of DAMP release. Apoptosis is non-lytic: executioner caspases-3/-7 dismantle the cell into membrane-enclosed apoptotic bodies, phosphatidylserine exposure serves as an “eat-me” signal, and DAMPs are not passively released through membrane rupture but may be actively secreted in small quantities (e.g., ATP via pannexin-1 channels, IL-33) as “find-me” signals for phagocytic clearance [27,28]. Only when phagocytic clearance fails, as may occur during massive heatstroke-induced tissue injury, apoptotic cells progress to secondary necrosis, a passive process that breaches the membrane and releases the full DAMP repertoire. By contrast, pyroptosis (GSDMD pore-mediated), necroptosis (MLKL pore-mediated), and ferroptosis (lipid peroxide-driven membrane failure) are all lytic: they actively disrupt plasma membrane integrity and release intracellular DAMPs including HMGB1, mtDNA, ATP, histones, and LDH into the extracellular space, driving the immunoinflammatory escalation described in Section 4.

Thermal injury does not trigger a single, uniform mode of cell death, but rather initiates a wide spectrum of RCD pathways whose predominance shifts depending on how intense the heat exposure is, how long it lasts, and how well the cell can sustain its energetic and proteostatic balance [30]. Under milder conditions, cells often begin with apoptosis, a controlled and relatively orderly process aimed at containing damage. However, as thermal stress intensifies and the cell’s capacity to maintain protein folding declines, chaperone systems become saturated and mitochondrial function deteriorates. In this progressively destabilized environment, apoptosis can give way to more destructive and inflammatory forms of cell death [31]. Heat-induced injury therefore unfolds along a continuum rather than as isolated mechanisms, transitioning from apoptosis to pyroptosis, ferroptosis, necroptosis, and finally unregulated necrosis. Each step along this continuum contributes in a distinct way to tissue injury, whether by releasing inflammatory DAMPs, damaging endothelial structures, or promoting microvascular dysfunction, and together these processes set the stage for the coagulopathy and multiorgan complications characteristic of severe heatstroke [2].

As indicated by recent research, controlled cell death pathways are linked and overlapping reactions to extreme cellular stress rather than traditionally being understood as separate molecular programs. In this regard, PANoptosis, a newly identified inflammatory cell death process, integrates essential elements of necroptosis, apoptosis, and pyroptosis into a coordinated signaling network. PANoptosis represents the creation of multiprotein PANoptosome complexes that can concurrently activate caspases, inflammasome signaling, and RIPK-mediated necrotic pathways, in contrast to the conventional belief that these pathways function independently. The exact role of PANoptosis in heatstroke-induced coagulopathy and MODS is yet unknown because this research is still in its infancy. However, the significant overlap between controlled cell death pathways during advanced thermal injury may be explained by adding PANoptosis to the existing paradigm. For conceptual clarity, apoptosis, pyroptosis, ferroptosis, and necroptosis are treated separately; however, these routes are not distinct processes. These types of controlled cell death exhibit significant molecular interaction, and their relative contributions probably differ depending on the type of tissue, the severity of the injury, and the temporal stage. Multiple RCD pathways may be activated simultaneously in advanced heat-induced damage, which gives rise to new ideas like PANoptosis [32].

### 3.1. Apoptosis: The Early, Energy-Dependent Response

Apoptosis is one of the earliest and best-characterized regulated cell death pathways observed under sublethal heat stress; however, current evidence does not support a universally conserved temporal sequence among apoptosis, pyroptosis, ferroptosis, and necroptosis, which may coexist and interact through extensive molecular crosstalk. Mitochondrial perturbations, including loss of membrane potential, cytochrome c release, and apoptosome formation, drive caspase-9 and downstream caspase-3 activation, enabling a controlled dismantling of damaged cells. This route depends on ATP availability and intact proteostasis, which explains why apoptosis predominates during moderate hyperthermia, when HSP70 and other chaperones still buffer protein unfolding [32,33]. Increased apoptotic signaling, which is characterized by enhanced caspase-3 activation, cytochrome c release, and mitochondrial membrane depolarization after acute hyperthermia, has been shown in intestinal, renal, and neural tissues in experimental heatstroke models. DNA fragmentation and elevated Bax/Bcl-2 ratios have also been seen in heat-stressed tissues, indicating that mitochondrial apoptosis plays a part in the development of thermal injury [34].

Heat-stressed keratinocytes also engage extrinsic apoptosis, where death receptors (Fas, TNFR1, TRAIL-R) activate caspase-8. Crosstalk between extrinsic and intrinsic branches occurs through BID cleavage, amplifying mitochondrial permeabilization.

The intrinsic apoptotic pathway is triggered by intracellular stress signals that activate BH3-only proteins, including BIM, BID, PUMA, and NOXA. These proteins antagonize pro-survival BCL-2 family members, facilitating BAX/BAK oligomerization at the outer mitochondrial membrane and promoting mitochondrial outer membrane permeabilization (MOMP). Subsequent release of cytochrome c into the cytosol enables the assembly of the apoptosome through interaction with APAF-1 and procaspase-9, leading to activation of caspase-9 and downstream executioner caspases-3 and -7. The resulting proteolytic cascade drives characteristic apoptotic changes, including cell shrinkage, chromatin condensation, DNA fragmentation, and formation of membrane-enclosed apoptotic bodies. Notably, apoptosis does not occur in isolation and may coexist with other forms of regulated cell death, including pyroptosis, ferroptosis, and necroptosis, which share overlapping stress signals and exhibit extensive molecular crosstalk. Depending on the cellular context and severity of injury, these pathways may be activated simultaneously or sequentially, with no universally established temporal hierarchy. The apoptotic pathway can be modulated pharmacologically through BCL-2-targeting agents, BH3 mimetics, or pan-caspase inhibitors such as zVAD-fmk [35].

In heatstroke animal models, intrinsic apoptosis has been directly demonstrated in intestinal epithelial cells and hippocampal neurons. Heat stress induces intestinal injury through a lysosomal–mitochondrial apoptotic pathway, characterized by an increased Bax/Bcl-2 ratio, cytochrome c release, and activation of caspase-9 and caspase-3 both in vivo and in vitro; pre-treatment with N-acetyl-L-cysteine (NAC) inhibited ROS generation and suppressed the entire cascade, confirming oxidative stress as the upstream trigger [36].

### 3.2. Pyroptosis: Caspase-Driven Inflammatory Lysis

As heat-induced injury accelerates and DAMP-PRR signaling intensifies, cells may shift toward pyroptosis, an inflammatory cell death mediated by caspase-1 (canonical) or caspase-4/5/11 (non-canonical). Activated inflammatory caspases cleave gasdermin D (GSDMD), whose N-terminal fragments oligomerize in the plasma membrane to form large pores, triggering osmotic lysis and massive release of IL-1β and IL-18 [37].

Hyperthermia, oxidative stress, and intestinal barrier breakdown all enhance NLRP3 inflammasome priming, making pyroptosis a key contributor to systemic inflammation, endothelial activation, and leukocyte dysfunction in severe heat injury. Pyroptosis marks the point at which cell death becomes actively pro-inflammatory, amplifying microvascular damage. Pyroptosis has been directly demonstrated in multiple tissues in heatstroke models [38]. A mechanistic role for pyroptosis in hyperthermia-induced inflammatory tissue injury has been supported by recent studies in heatstroke-associated acute lung and intestinal injury, which have shown activation of the NLRP3 inflammasome pathway, increased caspase-1 cleavage, gasdermin D activation, and elevated IL-1β and IL-18 release showed that heatstroke induces hepatocyte pyroptosis characterized by activated caspase-1, cleaved IL-1β, and elevated ROS-NLRP3 signaling in a rat heatstroke model; the Ang-(1-7) analog AVE 0991 attenuated both pyroptosis and liver damage by inhibiting the ROS-NLRP3 pathway [39].

### 3.3. Ferroptosis: Iron-Dependent Lipid Peroxidation

As mitochondrial dysfunction deepens and antioxidant systems fail, heat stress promotes ferroptosis, an iron-driven process characterized by catastrophic lipid peroxidation of polyunsaturated phospholipids. This occurs when glutathione (GSH) depletion and GPX4 inactivation impair the cell’s ability to detoxify lipid hydroperoxides. Ferroptosis is tightly linked to mitochondrial ROS overproduction, imbalanced iron handling, and failure of proteostasis, the last of which increases susceptibility to oxidative membrane collapse [40].

Experimental models of hyperthermia show that ferroptosis is a prominent driver of lung, intestinal, and renal damage in heatstroke. Further evidence of ferroptotic injury, especially in renal and pulmonary tissues, has been shown using heat stress models, including iron-dependent membrane damage, glutathione depletion, GPX4 downregulation, and lipid ROS buildup. In models of hyperthermia, experimental ferroptosis inhibition has been linked to a partial reduction in tissue damage and oxidative damage [41].

Emerging evidence indicates extensive crosstalk among major regulated cell death pathways, including autophagy, apoptosis, ferroptosis, and necroptosis, particularly under conditions of severe oxidative and mitochondrial stress. Autophagy is tightly linked to apoptosis through the Bcl-2/Beclin-1 regulatory axis and caspase-mediated cleavage events, while ferroptosis is functionally interconnected with autophagic flux via ferritinophagy (NCOA4-dependent iron release), thereby modulating intracellular iron availability and lipid peroxidation. In parallel, apoptosis and necroptosis are reciprocally regulated through caspase-8-dependent molecular switching mechanisms. Mitochondria-derived reactive oxygen species act as a central upstream hub that coordinates the activation of these distinct but interrelated cell death programs. Despite these well-established molecular links, an integrated framework that unifies autophagy-, apoptosis-, ferroptosis-, and necroptosis-related signaling into a single stress-responsive cell death network remains incompletely defined, particularly in the context of heat- or injury-induced neuronal stress responses [42,43,44].

### 3.4. Necroptosis: RIPK-Driven Programmed Necrosis

When apoptotic caspases (particularly caspase-8) are inhibited or overwhelmed, cells may enter necroptosis, a programmed necrotic pathway orchestrated by RIPK1, RIPK3, and their downstream effector MLKL. RIPK1/RIPK3 oligomerization forms the necrosome, enabling MLKL phosphorylation and translocation to the plasma membrane, where it generates disruptive pores leading to cell swelling and rupture.

Necroptosis is especially relevant in heatstroke because TNF-α levels rise rapidly during hyperthermia while the inflammatory microenvironment suppresses caspase-8 activity, the combination of conditions that favors necrosome assembly. Once engaged, necroptosis magnifies vascular thrombosis and endothelial injury.

Animal studies demonstrate that necroptosis inhibitors (e.g., necrostatin-1) can reduce microvascular thrombosis and improve survival, highlighting its clinical significance [45].

Necroptosis has been demonstrated across multiple tissue types in heatstroke models. In pulmonary microvascular endothelial cells (PMVECs), heat stress induces RIP1 and MLKL upregulation and RIP1–RIP3 necrosome formation, driving HMGB1 nuclear-to-cytoplasmic translocation via ERK, NF-κB, and c-Jun signaling; necrostatin-1 and siRNA-mediated RIP1/RIP3 knockdown attenuate both necroptosis and HMGB1 release. In intestinal epithelial cells and a C57BL/6 heatstroke mouse model, heat activates a p53-dependent TLR3–TRIF–RIP3–MLKL pathway; p53 knockout and pifithrin-α block TRIF–RIP3 complex formation and intestinal necroptosis in vivo. Most significantly, in a heatstroke mouse model, ZBP1 directly activates RIPK3–MLKL-dependent necroptosis specifically in myeloid cells, triggering tissue factor release and phosphatidylserine externalization that drive disseminated intravascular coagulation, multiorgan injury, and mortality, as confirmed by ZBP1, RIPK3, and MLKL knockout mice, conditional myeloid-lineage deletion, and bone marrow chimera experiments. Collectively, these studies establish necroptosis as a causal contributor to heatstroke pathology across endothelial, epithelial, vascular, and myeloid compartments, with direct mechanistic links to both DAMP-driven inflammation and coagulopathy [32,46].

### 3.5. Necrosis: The Terminal, Unregulated End Stage

At extreme temperatures, ATP reserves collapse, membrane pumps fail, and cells undergo necrosis, the unregulated form of cell death. This stage reflects the final loss of all adaptive and regulated mechanisms. Thermal necrosis drives the zone of coagulation in burn injuries and promotes rapid propagation of inflammatory and coagulopathic cascades. Severe heatstroke has also been linked to necroptotic signaling, as evidenced by enhanced RIPK1, RIPK3, and MLKL activation in experimental thermal injury models. Significantly, necrostatin-1 pharmacologic suppression has been demonstrated to decrease microvascular thrombosis, inflammatory amplification, and endothelial damage, indicating a possible mechanistic role for necroptosis in heatstroke-associated coagulopathy [32,46,47].

Although these pathways are biologically interconnected and may coexist during severe hyperthermia, they differ substantially in their activation mechanisms, inflammatory potential, molecular mediators, and biological consequences. A comparative overview of the principal characteristics of major heat stress-associated cell death pathways is presented in Table 1.

### 3.6. A Unified Continuum: Shifting Modes of Cell Death Under Heat Stress

**Figure 2:**
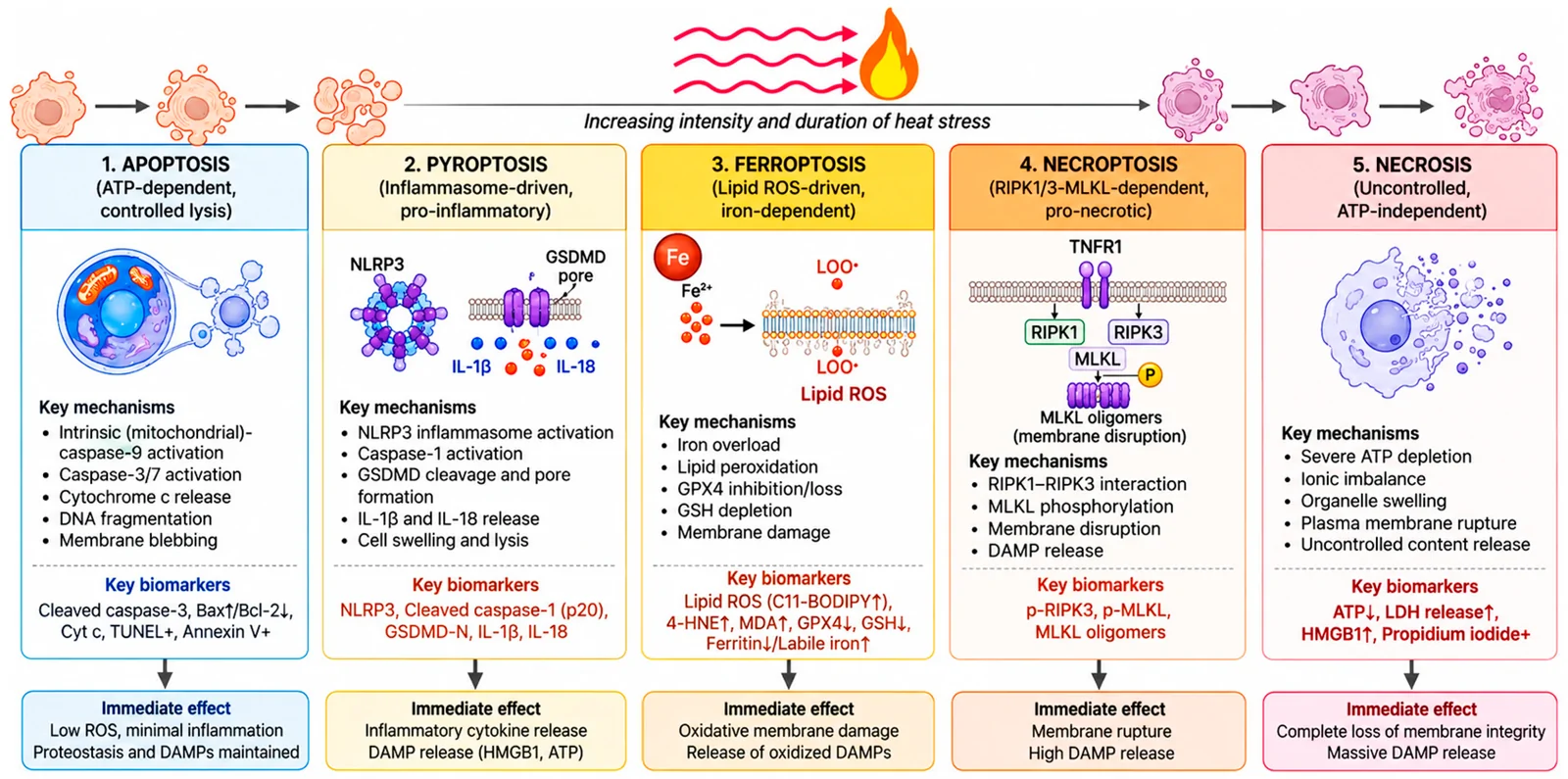
Demonstrates the progression of heat-induced regulated cell death (RCD) pathways from damage that is reversible to damage that is irreversible. Cells gradually transition from adaptive and perhaps reversible responses to irreversible damage as heat stress intensity and duration increase. Apoptosis, which maintains membrane integrity and restricts inflammatory signals, is the main characteristic of the early stages. Pyroptosis is encouraged by inflammasome activation in response to increasing oxidative and proteotoxic stress, whereas ferroptosis is driven by iron-dependent lipid peroxidation. Necroptosis and eventually unchecked necrosis become more prevalent when there is significant cellular damage and ongoing inflammatory signals. Although each pathway has unique molecular mediators, biomarkers, and direct cellular effects, they all lead to the production of DAMP, inflammation, endothelial damage, microvascular dysfunction, and the development of multiorgan failure. The diagram is intended as a conceptual representation of interconnected pathogenic mechanisms and does not imply a strictly sequential or universally conserved temporal progression of regulated cell death pathways. Apoptosis, pyroptosis, ferroptosis, and necroptosis may occur concomitantly, overlap temporally, and exhibit substantial molecular crosstalk depending on the cellular context and severity of thermal injury. Created in BioRender. Ajanovic, T. (2026) https://BioRender.com/zcx8wb6.

Heat-induced injury therefore unfolds as a dynamic network of interconnected regulated cell death pathways rather than as isolated mechanisms. Apoptosis, pyroptosis, ferroptosis, and necroptosis may be activated concurrently, partially overlap, or predominate at different stages of injury depending on cellular context, metabolic status, and the intensity and duration of thermal stress. Figure 2 illustrates how increasing thermal stress may alter the relative contribution of different regulated cell death pathways within a dynamic and interconnected network of cellular responses. Under moderate and reversible heat exposure, cells primarily engage apoptosis, an ATP-dependent and orderly process that preserves membrane integrity and limits inflammatory spillover. As thermal stress intensifies, pyroptosis may emerge through inflammasome activation and gasdermin-mediated pore formation, promoting IL-1β and IL-18 release. With more severe heat and rising oxidative burden, cells transition toward ferroptosis, characterized by lipid ROS accumulation and iron-dependent membrane damage. This ordered progression, however, describes only the dominant tendency at increasing thermal loads; in practice these modalities are not sequential stages but interconnected programs that share molecular machinery and frequently operate in parallel. Several molecular nodes explain why the modalities overlap rather than succeed one another. Caspase-8 functions as a central switch: in its active state it executes apoptosis while simultaneously cleaving RIPK1 and RIPK3 to restrain necroptosis, so that conditions suppressing caspase-8 activity, such as the inflammatory, ROS-rich microenvironment of severe heatstroke, redirect the same upstream signal toward necroptotic death. RIPK1 likewise acts as a hub whose kinase activity determines whether a cell survives, undergoes apoptosis, or commits to necroptosis. Crosstalk extends to the lytic pathways as well: executioner caspase-3 can cleave gasdermin E, converting an ostensibly silent apoptotic program into secondary pyroptosis in cells expressing this gasdermin. Which modality predominates in a given cell at a given moment is governed by the same variables that the continuum model places on its axes. ATP availability is decisive: apoptosis and the assembly of the apoptosome are energy-dependent, so a cell that retains sufficient ATP tends toward apoptosis, whereas bioenergetic collapse forecloses this option and shifts the balance toward necroptosis and necrosis. Mitochondrial function acts in the same direction, since loss of membrane potential not only curtails ATP synthesis but also amplifies ROS output and releases pro-apoptotic and pro-inflammatory mediators. Oxidative stress lowers the threshold for ferroptosis and pyroptosis in parallel, as accumulating lipid peroxides drive ferroptotic membrane failure while mitochondrial ROS prime the NLRP3 inflammasome. The inflammatory status of the microenvironment further biases the outcome: a TNF-α-rich, caspase-8-suppressed milieu favors necroptosis, while abundant DAMP–PRR signaling reinforces pyroptosis. Iron availability and glutathione/GPX4 capacity specifically gate ferroptotic susceptibility. Because severe hyperthermia perturbs all of these variables at once, neighboring cells, and even subcellular regions within a single cell, may cross different death thresholds concurrently, producing the mixed and overlapping death signatures characteristic of heat-injured tissue [51,52,53].

Finally, these modalities overlap not only in molecular terms but also in time and space. Rather than the tissue passing cleanly through one death program before the next begins, the dominant modality shifts dynamically as local conditions evolve: an early, relatively synchronized wave of apoptosis in moderately stressed cells gives way to, and increasingly coexists with, lytic death as ATP is depleted, oxidative burden accumulates, and DAMP-driven inflammation intensifies. At any given moment the injured tissue therefore contains cells at different points along the continuum, so that apoptotic, pyroptotic, ferroptotic, and necroptotic signatures are detected concurrently rather than in strict succession. This temporal and spatial heterogeneity is itself self-amplifying, because DAMPs released by the earliest dying cells lower the death threshold in their neighbors and bias them toward the more inflammatory lytic modalities. Viewed this way, the continuum is best understood not as a fixed linear sequence but as a dynamic, overlapping landscape whose local composition reflects the momentary balance of energy charge, redox state, and inflammatory signaling, of which PANoptosis represents the most fully integrated expression [37,38,39].

### 3.7. Pathophysiological Implications

As cells progress along this continuum of heat-induced injury, their behavior changes in a way that amplifies damage at every step.

Given that regulated cell death pathways differ substantially in their molecular mechanisms, inflammatory potential, and contribution to endothelial injury and coagulopathy, emerging therapeutic strategies should also be interpreted according to the specific pathways they target. Rather than representing isolated interventions, many of these approaches aim to modulate distinct components of the heat stress–RCD–inflammation axis, including proteostasis failure, oxidative stress, inflammasome activation, ferroptotic lipid peroxidation, necroptotic signaling, and endothelial dysfunction. A categorized overview of current and emerging therapeutic strategies according to their principal targeted cell death pathways and mechanisms is presented in Table 2.

## 4. DAMP Release and Immunoinflammatory Escalation

Heat-induced proteostasis collapse and RCD create the intracellular conditions that promote the release of DAMPs, endogenous danger signals that alert and activate the innate immune system. DAMP release marks the transition from cell-autonomous stress to tissue-level inflammation, initiating a cascade that ultimately drives endothelial dysfunction, glycocalyx degradation, microvascular thrombosis, and systemic inflammation characteristic of severe heatstroke and heat-related multiorgan failure [59,60]. An important unresolved question in heatstroke pathophysiology is whether the inflammatory response primarily acts as a driver of tissue injury or represents a secondary amplification mechanism following cellular damage. While DAMP-mediated signaling clearly contributes to injury propagation, current evidence suggests that inflammation may arise as a consequence of cellular stress responses, proteostasis disruption, and regulated cell death; however, the precise causal hierarchy among these events remains incompletely established.

A key unresolved question concerns whether DAMPs should be viewed as primary pathogenic drivers or as secondary amplifiers of heatstroke-associated injury. Based on the mechanistic framework proposed in this review, the available evidence more strongly supports the latter interpretation. Heat-induced protein denaturation, proteostasis disruption, mitochondrial dysfunction, and activation of regulated cell death pathways generally precede substantial extracellular DAMP accumulation. Consequently, DAMP signaling appears most likely to function as a potent amplification mechanism that propagates and sustains inflammation once cellular injury has already been initiated. Nevertheless, reciprocal interactions between DAMP-mediated signaling and cellular stress pathways may establish self-reinforcing feedback loops that further exacerbate tissue damage and systemic organ dysfunction.

### 4.1. Origins and Classes of DAMPs in Heat Injury

Accordingly, DAMPs occupy an intermediate position within the proposed pathogenic cascade. They are unlikely to represent the earliest molecular trigger of heatstroke injury but instead serve as critical mediators linking local cellular damage to systemic immunoinflammatory escalation. This distinction has important therapeutic implications, as interventions targeting DAMP signaling may be expected to attenuate propagation of injury rather than prevent the initial thermal insult itself.

Thermal stress compromises the integrity of multiple cellular compartments, each of which releases a characteristic set of DAMPs upon failure (Table 3). Severe hyperthermia, oxidative stress, and the various forms of RCD, apoptosis, necroptosis, pyroptosis, and ferroptosis, accelerate this release, ranging from the controlled secretion seen in apoptosis to the catastrophic spillage of lytic cell death. The cellular origin and receptor targets of the principal DAMP classes are summarized in Table 3.

Once outside the cell, many of these molecules act as powerful pro-inflammatory mediators. They engage pattern recognition receptors such as TLR2, TLR4, RAGE (AGER), and members of the NOD-like receptor family, activating signaling pathways that rapidly escalate the innate immune response. In this way, DAMPs serve as the critical molecular link between intracellular injury and the widespread inflammation that characterizes severe heat-induced pathology. Thermal stress promotes the release of multiple DAMP classes originating from the nucleus, mitochondria, cytosol, endoplasmic reticulum, and extracellular matrix, which subsequently activate pattern-recognition receptors including TLRs, RAGE, and inflammasome-associated [44,61].

**Table 3:** Classes of Heat-Induced DAMPs, Cellular Origin, and PRR Targets.

Cellular Compartment	Representative DAMPs	Primary PRRs Activated	Functional Consequences	References
Nucleus	HMGB1; histones; IL-33; profilin-1	TLR2; TLR4; RAGE	Strong NF-κB activation; endothelial permeability; cytokine surge	[62]
Mitochondria	mtDNA; ATP; cytochrome-c; formyl peptides	TLR9; NLRP3; cGAS-STING	Inflammasome activation; IL-1β/IL-18 maturation; pyroptosis	[63]
Endoplasmic Reticulum	Calreticulin	CD91; scavenger receptors	Immunogenic cell death signaling; phagocyte recruitment	[64]
Cytosol	Ferritin; S100 proteins	RAGE; TLR4	Amplification of inflammation; chemotaxis	[65]
Cytoplasmic Granules	LL-37; antimicrobial peptides	FPR2; TLRs	Neutrophil activation; chemokine amplification	[66]
Extracellular Matrix	Hyaluronan fragments; fibronectin; matrilin-2; enolase-1	TLR2; TLR4	Matrix degradation; leukocyte adhesion; endothelial activation	[67]

### 4.2. PRR Activation and Inflammasome Signaling

DAMPs play a central role in amplifying the inflammatory response that follows heat-induced cellular injury (Table 4). Once released into the extracellular environment, these molecules interact with a wide range of pattern recognition receptors expressed on neutrophils, monocytes, macrophages, dendritic cells, and endothelial cells, thereby converting localized cellular stress into a coordinated systemic reaction. Among the most important of these receptors are the Toll-like receptors, particularly TLR2 and TLR4, which are key drivers of both burn-related and heat-related inflammation. Their engagement triggers NF-κB activation, leading to the transcription of major pro-inflammatory cytokines, including IL-1β, IL-6, IL-8, TNF-α, and a broad array of chemokines that sustain and amplify the inflammatory cascade. Notably, experimental inhibition of these receptors has been shown to reduce systemic inflammation and lessen organ injury [68,69].

The immunoinflammatory impact of regulated cell death in heatstroke is not determined simply by whether DAMPs are released, but by which RCD modality produces them, because each pathway generates a qualitatively and quantitatively distinct DAMP signature with different downstream consequences. At one end of the spectrum, apoptosis actively neutralizes its own DAMPs: caspase-mediated cleavage of complex I subunit p75/NDUFS1 in permeabilized mitochondria generates ROS that oxidizes a critical cysteine residue on HMGB1, converting it from its pro-inflammatory reduced form to an immunologically silent oxidized form that promotes tolerance rather than immunity [77,78].

At the opposite end, necroptosis generates a particularly immunostimulatory DAMP repertoire. The absence of caspase activity preserves DAMPs in their pro-inflammatory forms: HMGB1 remains in its reduced all-thiol state (chemotactic via CXCR4) or disulfide form (cytokine-inducing via TLR4), full-length IL-33 is released intact, and high-molecular-weight genomic DNA is expelled within intact nuclei. Ferroptosis is lytic, though its terminal membrane rupture also requires NINJ1 oligomerization. The upstream mechanism, however, differs fundamentally from pyroptosis and necroptosis, being driven by iron-dependent lipid peroxidation rather than dedicated protein pore formation. Dying ferroptotic cells release intracellular contents including DAMPs that can trigger innate immune activation. Moreover, adding ferroptotic cells to immunogenic apoptotic cells actively dampened their vaccination potential, indicating that ferroptosis exerts a net immunosuppressive effect on adaptive immunity [79,80].

Pyroptosis is highly inflammatory. Before terminal lysis, GSDMD pores allow for controlled release of small DAMPs and mature IL-1β/IL-18, which serve as potent pro-inflammatory mediators. Ca^2+^ influx through GSDMD pores also activates tissue factor, directly linking pyroptosis to coagulation. Upon NINJ1-mediated terminal rupture, larger DAMPs (HMGB1, LDH) are released. In heatstroke, pyroptosis-derived IL-1β and IL-18 are key drivers of the cytokine storm that precedes multiorgan failure [81].

Importantly, the immunological consequences of regulated cell death are highly context-dependent and cannot be attributed solely to the molecular death pathway involved. The inflammatory outcome is influenced by multiple variables, including the affected cell type, tissue microenvironment, local cytokine composition, oxidative stress burden, endothelial activation status, and the efficiency of phagocytic clearance mechanisms. For example, apoptotic death of epithelial cells that are rapidly removed by resident phagocytes may remain largely immunologically silent, whereas defective clearance during massive tissue injury can result in secondary necrosis and substantial DAMP release. Likewise, pyroptosis occurring in macrophages may generate a markedly different inflammatory signature compared with pyroptosis in endothelial, epithelial, or parenchymal cells because of differences in cytokine production, inflammasome activity, and downstream immune-cell recruitment. Similar considerations apply to ferroptosis and necroptosis, whose immunological effects vary according to local redox conditions, iron availability, tissue-specific DAMP repertoires, and interactions with infiltrating immune cells. Furthermore, the inflammatory consequences of cell death may evolve over time, with early-stage signaling events differing substantially from those associated with late membrane rupture, secondary DAMP release, and amplification of thromboinflammatory responses. Therefore, the classifications presented in this review should be interpreted as general mechanistic tendencies rather than fixed or universally applicable immunological outcomes [82,83,84,85,86,87,88,89,90,91].

To clarify the strength of evidence underlying the DAMP/PRR/inflammasome axis in heat-related injury, we graded the principal pathways by their source of support. Table 5 distinguishes data derived from human heatstroke, animal heatstroke or hyperthermia models, in vitro hyperthermia studies, and extrapolation from burn injury, sepsis, and other sterile inflammatory conditions. Because much of the mechanistic literature on DAMP signaling originates from burn injury, sterile inflammation, sepsis, and ischemia–reperfusion models, caution is required when extrapolating these findings to heatstroke. While many downstream mediators appear shared across these conditions, their relative contribution, timing of activation, and pathophysiological significance in heatstroke remain incompletely defined.

DAMP-PRR signaling may directly contribute to the development of heatstroke-induced coagulopathy in addition to cytokine amplification. Endothelial activation, tissue factor production, NF-κB-dependent inflammatory responses, and glycocalyx breakdown are all facilitated by the activation of TLR4 and NLRP3 signaling pathways in immune and endothelial cells. A pro-coagulant endothelial phenotype marked by elevated leukocyte adhesion, platelet activation, microvascular thrombosis, and compromised anticoagulant function is favored by these alterations.

Importantly, this pathway provides a direct mechanistic link between sterile inflammation and coagulopathy in heatstroke, whereby DAMP-mediated TLR4/NLRP3 activation drives endothelial transition toward a pro-coagulant phenotype characterized by tissue factor expression, glycocalyx degradation, and microvascular thrombosis.

### 4.3. Cytokine Storm: Amplification of Systemic Inflammation

Cytokines increase endothelial permeability, promote the recruitment of leukocytes into injured tissues, and accelerate coagulation pathways. Additional chemotactic cytokines, such as IL-8 and MCP-1, further intensify inflammation by driving sustained infiltration of neutrophils and monocytes into vulnerable tissues [104].

In parallel, IL-10, an anti-inflammatory cytokine, rises early as part of the body’s attempt to counterbalance excessive inflammation. However, when present at persistently high levels, it becomes maladaptive, contributing to immune suppression and increasing susceptibility to sepsis. Importantly, the cytokine profile does not merely reflect the extent of injury but also reveals features of the host immune phenotype. Studies show that adults exhibit more pronounced IL-6 and IFN-γ responses than children, highlighting age-related differences in the inflammatory reaction to severe heat stress [97,103].

### 4.4. Neutrophil and Monocyte Activation: The Frontline of Hyperinflammation

Once released into injured tissues, DAMPs act as powerful activators of the innate immune system, rapidly mobilizing neutrophils as first responders. These cells infiltrate the damaged area in large numbers and unleash a broad arsenal of effector molecules, including ROS, proteases, matrix metalloproteinases, and neutrophil extracellular traps (NETs). NETs are particularly consequential, as their web-like DNA–protein structures not only trap pathogens but also provide a scaffold that promotes thrombosis, thereby intensifying endothelial injury and accelerating coagulation activation. Monocytes and macrophages arrive shortly thereafter, responding to the same DAMP signals. These cells serve a dual role: they amplify inflammation by producing key cytokines such as TNF-α, IL-1β, and IL-6, while simultaneously releasing IL-10 and other mediators involved in resolving inflammation and initiating tissue repair. When this delicate balance is disrupted, either by persistent thermal stress or overwhelming inflammatory signaling, the transition from injury to repair becomes impaired. The result is prolonged inflammation, delayed wound healing, and heightened vulnerability to secondary infections [100,105]. Systemically, these events are reflected in characteristic changes in blood profiles. Neutrophils and platelets become increasingly dominant, while lymphocyte counts fall, producing a pattern that signals sustained innate immune activation coupled with a weakening of adaptive immunity, a hallmark of severe heat-induced systemic stress [98,100].

## 5. Endothelial Injury, Glycocalyx Breakdown, and the Pathogenesis of Heatstroke-Induced Coagulopathy

The vascular endothelium is one of the earliest and most critically affected targets of severe heat stress. Although only a single-cell-thick monolayer, the endothelium regulates vascular tone, permeability, hemostasis, and immune cell trafficking. As summarized in Table 6, under hyperthermic conditions, the combined effects of direct thermal toxicity, oxidative injury, inflammatory activation, and microcirculatory stress produce a rapid and profound shift toward a pro-inflammatory and pro-thrombotic endothelial phenotype, forming the mechanistic core of heatstroke-induced coagulopathy (HIC).

### 5.1. Glycocalyx Shedding: The First Structural Hit

The endothelial glycocalyx represents the first structural component to be compromised during hyperthermic stress. This fragile, carbohydrate-rich layer, comprising proteoglycans such as syndecans, long glycosaminoglycan chains, and a variety of adsorbed plasma proteins, plays a fundamental role in vascular homeostasis, acting as both a permeability regulator and an anti-adhesive barrier. Its architecture is exquisitely sensitive to oxidative stress and enzymatic degradation. During hyperthermia, the excessive production of ROS weakens glycosaminoglycan side chains, while the heat-induced activation of sheddases, including matrix metalloproteinases and heparanases, accelerates proteoglycan cleavage. At the same time, pro-inflammatory cytokines such as TNF-α and IL-1β destabilize the glycocalyx matrix, further promoting its disintegration. As a result, circulating levels of syndecan-1 and hyaluronan rise rapidly, serving as measurable biomarkers of glycocalyx loss and correlating strongly with endothelial barrier failure and clinical severity [2,4,6].

The erosion of this protective layer has immediate physiological consequences. Loss of the glycocalyx increases vascular permeability, exposes previously shielded adhesion molecules including ICAM-1 and VCAM-1, and thereby predisposes the activated endothelium to enhanced leukocyte adhesion and subsequent microvascular thrombosis.

### 5.2. Oxidative Stress and Impaired Nitric Oxide Homeostasis

As hyperthermia progresses, oxidative stress becomes a dominant contributor to endothelial dysfunction. Mitochondrial impairment, activation of NADPH oxidase, and inflammatory cascades all amplify ROS production. These oxidants inflict direct biochemical damage by modifying membrane lipids and intracellular proteins, while also exerting critical effects on nitric oxide (NO) signaling. Excessive ROS rapidly scavenge NO, decrease its bioavailability, and impair endothelial NO synthase (eNOS) activity. In addition, redox-sensitive transcription factors such as NF-κB become activated, further fueling the inflammatory response.

The resulting deficit in NO disrupts vasodilatory capacity, increases vascular tone, and contributes to microcirculatory dysregulation, pathophysiologic changes intimately associated with heatstroke-induced shock. Endothelin-1, a potent vasoconstrictor, is frequently elevated under these conditions and exacerbates microvascular collapse, compounding the hemodynamic instability characteristic of severe heat stress [108,109].

### 5.3. Leukocyte Adhesion, Endothelial Activation, and Amplification Loops

**Figure 3:**
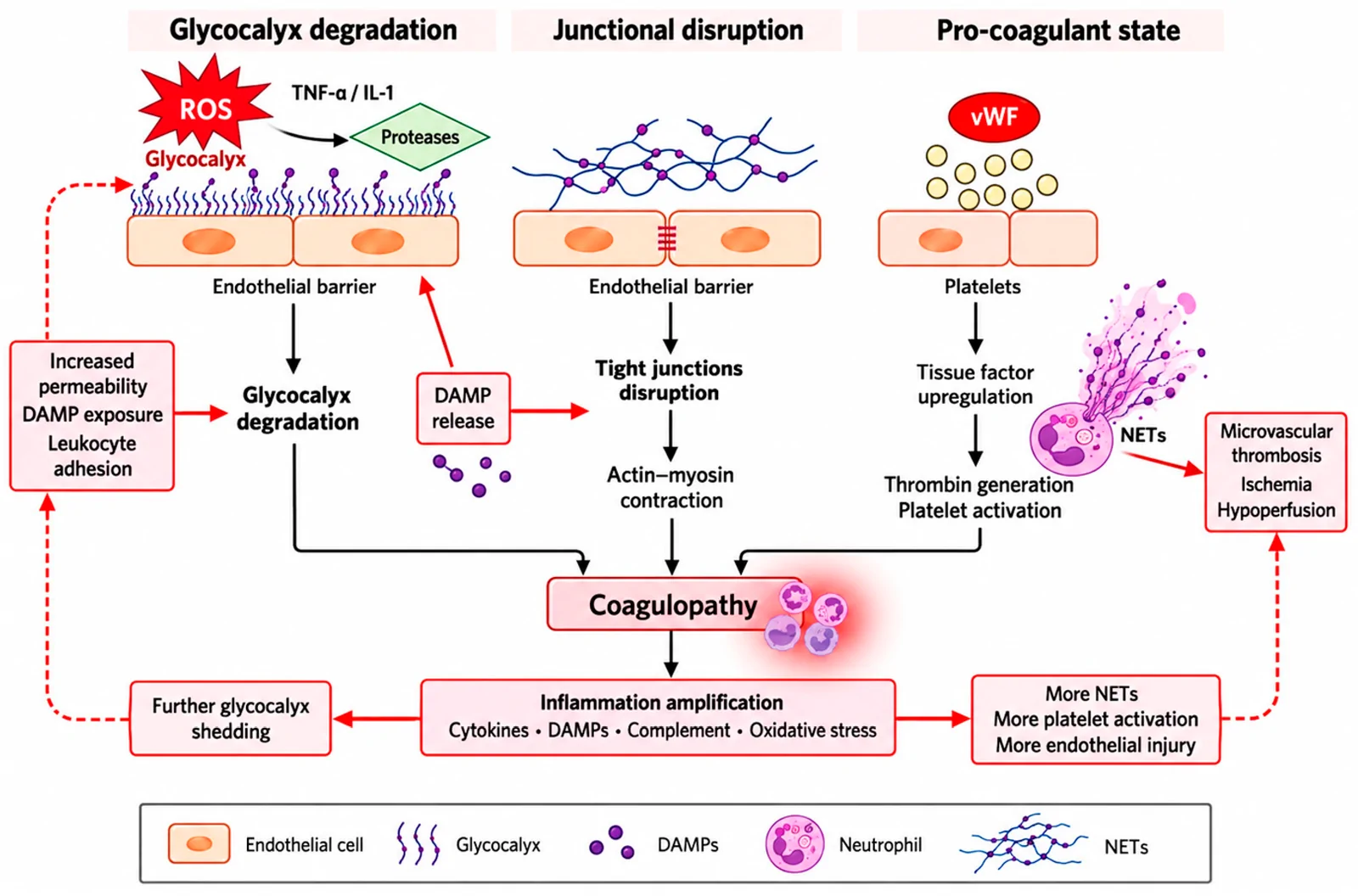
The mechanisms of endothelium damage resulting in coagulopathy caused by heatstroke. Multiple convergent routes of endothelial damage are triggered by severe heat. Protease activation, pro-inflammatory cytokines, and reactive oxygen species (ROS) all encourage the breakdown of glycocalyx, which increases vascular permeability, leukocyte adhesion, and DAMP release. Actin-myosin contraction and endothelial tight junction disruption both undermine barrier integrity and promote plasma leakage. Through von Willebrand factor release, platelet activation, tissue factor overexpression, and NET formation, endothelial stimulation further fosters a pro-coagulant phenotype that results in the production of thrombin and microvascular thrombosis. In the end, these mechanisms contribute to heatstroke-induced coagulopathy, tissue hypoperfusion, and organ failure by intensifying inflammation and endothelial damage in a self-sustaining loop. Created in BioRender. Ajanovic, T. (2026); https://BioRender.com/q2pg9xj.

With the glycocalyx stripped away, the endothelial surface becomes fully exposed and highly adhesive. Hyperthermia induces robust expression of ICAM-1, VCAM-1, and selectins, facilitating the firm adhesion and transmigration of neutrophils and monocytes. Once activated, neutrophils unleash a cascade of injurious mediators, including proteases, ROS, matrix metalloproteinases, and NETs. NETs are particularly damaging: they not only inflict direct cytotoxicity on the endothelium but also serve as structural scaffolds that promote platelet activation and microthrombus formation, thereby linking inflammatory activation to the development of coagulopathy. Monocyte–endothelial interactions further amplify this pathological loop [60,98,99]. These interactions stimulate additional cytokine production, enhance TF signaling, and contribute to progressive microvascular injury, creating a self-reinforcing cycle of endothelial activation and vascular damage (Figure 3).

### 5.4. Endothelial Activation and the Coagulation Switch: Mechanistic Basis of HIC

Endothelial injury shifts the hemostatic balance decisively toward a pro-coagulant phenotype. Activation of the TF pathway initiates thrombin generation, while the release of von Willebrand factor from Weibel–Palade bodies promotes platelet adhesion. At the same time, anticoagulant mechanisms become impaired through reduced thrombomodulin expression and diminished protein C activity. NETs continue to facilitate platelet recruitment and fibrin deposition within the microcirculation.

Multiple experimental observations highlight the centrality of endothelial dysfunction in heat-induced coagulopathy. Inhibition of the TF/FVIIa pathway attenuates thrombin generation, activated protein C has demonstrated endothelial-protective and survival-enhancing effects, and ZBP1–RIPK3-mediated cell death pathways directly contribute to microvascular thrombosis. Moreover, pharmacologic interventions such as sodium tanshinone IIA sulfonate have shown efficacy in reducing DIC and associated organ damage. Collectively, these findings confirm that heatstroke-associated coagulopathy is fundamentally endothelial-driven, arising from the intersection of thermal injury, inflammatory activation, and widespread microthrombotic occlusion [6,59]. Nevertheless, it should be noted that several supporting observations discussed above originate from burn, trauma, or other systemic inflammatory models. Although these studies consistently highlight endothelial dysfunction as a central determinant of thromboinflammatory injury, the quantitative contribution of individual endothelial pathways may differ in heatstroke and requires dedicated validation in heatstroke-specific experimental and clinical cohorts.

Crucially, it is important to consider glycocalyx shedding, tight junction disruption, and NET-mediated injury as parts of a self-amplifying endothelial injury loop rather than as separate occurrences. The endothelium surface is exposed to circulating inflammatory mediators, DAMPs, and activated leukocytes due to the first glycocalyx disintegration, which also increases endothelial permeability. Glycocalyx breakdown and endothelial dysfunction are further accelerated by the ensuing ischemia, oxidative stress, and inflammatory amplification, creating a positive feedback loop that leads to progressive vascular injury and heatstroke-induced coagulopathy. Mechanistically, this loop can be understood as a sequential and self-reinforcing cycle: initial glycocalyx degradation exposes adhesion molecules and increases endothelial permeability, facilitating leukocyte recruitment and activation; activated leukocytes release NETs and proteases that directly damage endothelial cells and promote platelet aggregation; concurrent DAMP release and PRR signaling further amplify NF-κB-mediated inflammation and tissue factor expression; microvascular thrombosis and impaired perfusion then exacerbate local hypoxia, oxidative stress, and endothelial injury, which in turn accelerates further glycocalyx shedding and barrier disruption, thereby closing and perpetuating the cycle [6,59,98].

### 5.5. Systemic Hemodynamic Collapse: Interplay of Vascular Leak and Microthrombosis

Once endothelial integrity is compromised, plasma extravasation ensues, leading to hemoconcentration and progressive intravascular volume depletion. Both relative and absolute hypovolemia reduce effective circulating volume and impair microvascular perfusion. The resulting ischemia-reperfusion–like injury further inflames tissues and promotes oxidative stress, perpetuating the cycle of endothelial damage and systemic inflammation. Microvascular thrombosis compounds these hemodynamic failures. Even in the presence of seemingly stable macrocirculatory parameters, oxygen delivery at the tissue level becomes critically impaired. This dissociation between systemic blood pressure and microcirculatory flow is a defining feature of heatstroke-induced vascular failure and contributes directly to MODS [6,59,108].

### 5.6. The Central Role of the Endothelium in Heatstroke Pathophysiology

Taken together, these mechanisms reveal the endothelium as the central integrator of heatstroke pathophysiology. Thermal injury initiates protein denaturation and an overwhelming burst of ROS; glycocalyx shedding rapidly increases permeability and promotes leukocyte adhesion; junctional breakdown allows for plasma leakage and tissue edema; endothelial activation triggers NF-κB signaling and upregulation of cytokines and adhesion molecules; a pronounced pro-coagulant shift develops through TF activation, vWF release, and NET-driven thrombosis; microcirculatory collapse ensues, leading to tissue hypoxia; and, ultimately, systemic involvement progresses toward MODS affecting the brain, kidneys, lungs, and liver. This integrative cascade underscores that endothelial-protective interventions, those capable of preserving glycocalyx integrity, stabilizing junctional complexes, and attenuating pro-coagulant signaling, represent promising therapeutic strategies in heatstroke and related burn-induced systemic inflammatory syndromes [6,59].

### 5.7. Heatstroke-Induced Coagulopathy and Endothelial Dysfunction: Distinction from Conventional DIC

Heatstroke-induced coagulopathy (HIC) is increasingly recognized as a distinct thromboinflammatory syndrome that shares several laboratory and clinical features with disseminated intravascular coagulation (DIC) but differs in its initiating mechanisms and pathophysiological drivers. In heatstroke, extreme hyperthermia directly injures endothelial cells and promotes systemic inflammation, resulting in activation of coagulation pathways, platelet consumption, and microvascular thrombosis. Endothelial injury represents a central event in HIC and precedes overt coagulation abnormalities in many patients [6,59]. The distinguishing features of heatstroke-induced coagulopathy (HIC) and conventional disseminated intravascular coagulation (DIC) are summarized in Table 7. Recent studies suggest that combining conventional coagulation tests with endothelial injury markers may improve early identification of patients at risk for severe HIC and multiorgan failure.

## 6. Systemic Consequences: MODS and Organ-Specific Damage

Severe heat exposure and extensive burn injury set off a profound, body-wide inflammatory and metabolic crisis that extends far beyond the initial site of tissue damage. As proteostasis collapse develops, intracellular stress accumulates and initiates regulated cell death pathways. This is followed by DAMP release and amplification of innate immune signaling. In parallel, endothelial dysfunction and microvascular thrombosis progressively impair tissue perfusion and oxygen delivery across multiple organs. When these pathological processes converge and escalate, they give rise to multiple organ MODS, the major determinant of mortality in both major burns and heatstroke [46,112] (Table 8).

### 6.1. MODS as the Final Common Pathway of Heat-Induced Injury

Clinical evidence shows that between one-third and one-half of adults with burns involving ≥20–40% of total body surface area ultimately develop severe MODS, typically beginning in the second post-burn week, often triggered or exacerbated by infection or sepsis. Mortality increases dramatically with each additional failing organ and approaches universality once three or more systems are compromised. Early metabolic indicators such as elevated lactate and base deficit are strongly associated with the later development of MODS and with poor overall outcomes. Experimental studies further reinforce this systemic perspective, demonstrating that even organs not directly exposed to heat, including the kidney, liver, lungs, cardiovascular system, intestine, and brain, show significant structural and functional injury, highlighting the truly global nature of thermal and burn-induced pathology. Rather than occurring as independent organ failures, MODS in heat injury reflects a progressive propagation of systemic inflammatory, metabolic, and microvascular dysfunction across interconnected organ systems [1,46,119,120].

### 6.2. Cardiovascular Dysfunction

The relative contribution of regulated cell death pathways may differ across organs and tissue contexts. For example, pyroptosis and necroptosis appear particularly relevant in immune and endothelial compartments where inflammatory signaling is dominant, whereas apoptosis and ferroptosis may play a more prominent role in metabolically active tissues such as the liver and kidney under thermal stress. However, direct comparative evidence in heatstroke remains limited, and the spatiotemporal distribution of RCD modalities across organ systems remains an important area for future research. Cardiac involvement emerges early after severe thermal injury and often persists throughout the clinical course. Patients typically experience an initial hyperdynamic phase, characterized by increased cardiac output and tachycardia, followed by a progressive decline in cardiac performance despite adequate fluid resuscitation [121,122].

Underpinning much of this dysfunction is mitochondrial injury, marked by oxidative stress, disruption of respiratory chain components, and ATP depletion. Cardiac dysfunction further exacerbates systemic hypoperfusion and inflammatory mediator accumulation, creating conditions that predispose the lungs to secondary injury and inflammatory overload.

### 6.3. Pulmonary and Airway Injury

Following early cardiovascular compromise, pulmonary injury becomes prominent due to combined effects of direct thermal/chemical insult and secondary inflammatory propagation through the systemic circulation. The lungs are therefore affected both as a target of inhalational injury and as a downstream organ exposed to circulating inflammatory mediators. Pulmonary injury, whether from heat, smoke, or both, is a major driver of morbidity and mortality. Thermal insult primarily affects the upper airway, causing mucosal swelling, ulceration, and potential obstruction within 24–36 h. Accumulation of airway casts composed of necrotic epithelium, fibrin, and mucus can worsen ventilation, leading to atelectasis or, when combined with systemic inflammation, progression to ARDS. Smoke-derived toxins such as carbon monoxide and hydrogen cyanide further impair oxygen utilization by blocking hemoglobin function and mitochondrial respiration. Pulmonary injury and impaired gas exchange contribute to systemic hypoxemia, which intensifies renal ischemia and increases susceptibility to acute kidney injury [8,9].

### 6.4. Acute Kidney Injury (AKI)

Renal dysfunction develops as a consequence of sustained hypoperfusion, systemic inflammation, and rhabdomyolysis, making the kidney particularly vulnerable to both early circulatory collapse and later toxic-inflammatory injury. AKI is one of the most frequent and deadly complications of major burns, with a pooled incidence of approximately 40%. Mortality ranges from 44% to over 80%, especially in patients with large TBSA involvement, inhalation injury, rhabdomyolysis, or early-onset renal failure [123,124].

Early-phase AKI is driven predominantly by hemodynamic disturbances, including hypovolemia caused by massive capillary leak, reduced cardiac output, and intense renal vasoconstriction mediated by catecholamines, RAAS activation, and vasopressin. In the later phase, AKI results from persistent systemic inflammation, sepsis, microvascular injury, oxidative stress, and exposure to nephrotoxins. Rhabdomyolysis and hemolysis further worsen renal injury through the release of myoglobin and hemoglobin, which cause tubular obstruction, oxidative damage, and renal vasoconstriction. Mortality in cases of rhabdomyolysis-associated renal failure can reach 50%. Renal dysfunction further amplifies systemic metabolic imbalance and inflammatory mediator accumulation, which can contribute to hepatic stress and impaired detoxification capacity [125,126].

### 6.5. Hepatic Dysfunction

In parallel with renal injury, hepatic dysfunction reflects both metabolic stress and systemic cytokine overload, leading to impaired acute-phase response and progressive failure of detoxification and synthetic function. Even without direct thermal insult, the liver exhibits early metabolic disruption, alterations in acute-phase protein synthesis, hepatic steatosis, and declines in complement proteins such as C3 and α2-macroglobulin [120]. Excessive cytokine exposure, particularly IL-6 and TNF-α, contributes to hepatocellular injury and impaired synthetic function, which in turn exacerbates systemic coagulopathy and immunologic dysfunction.

### 6.6. Intestinal Barrier Breakdown and Endotoxemia

Hyperthermia induces significant gastrointestinal injury, including intestinal ischemia, villus atrophy, epithelial shedding, and disruption of tight junctions. These structural changes increase permeability, allowing bacterial products such as LPS and additional DAMPs to enter the circulation, thereby contributing to endotoxemia and systemic inflammatory activation. Intestinal barrier breakdown and endotoxemia significantly amplify systemic inflammation, which facilitates blood–brain barrier disruption and central nervous system injury [108,112,127].

### 6.7. Central Nervous System Injury

**Figure 4:**
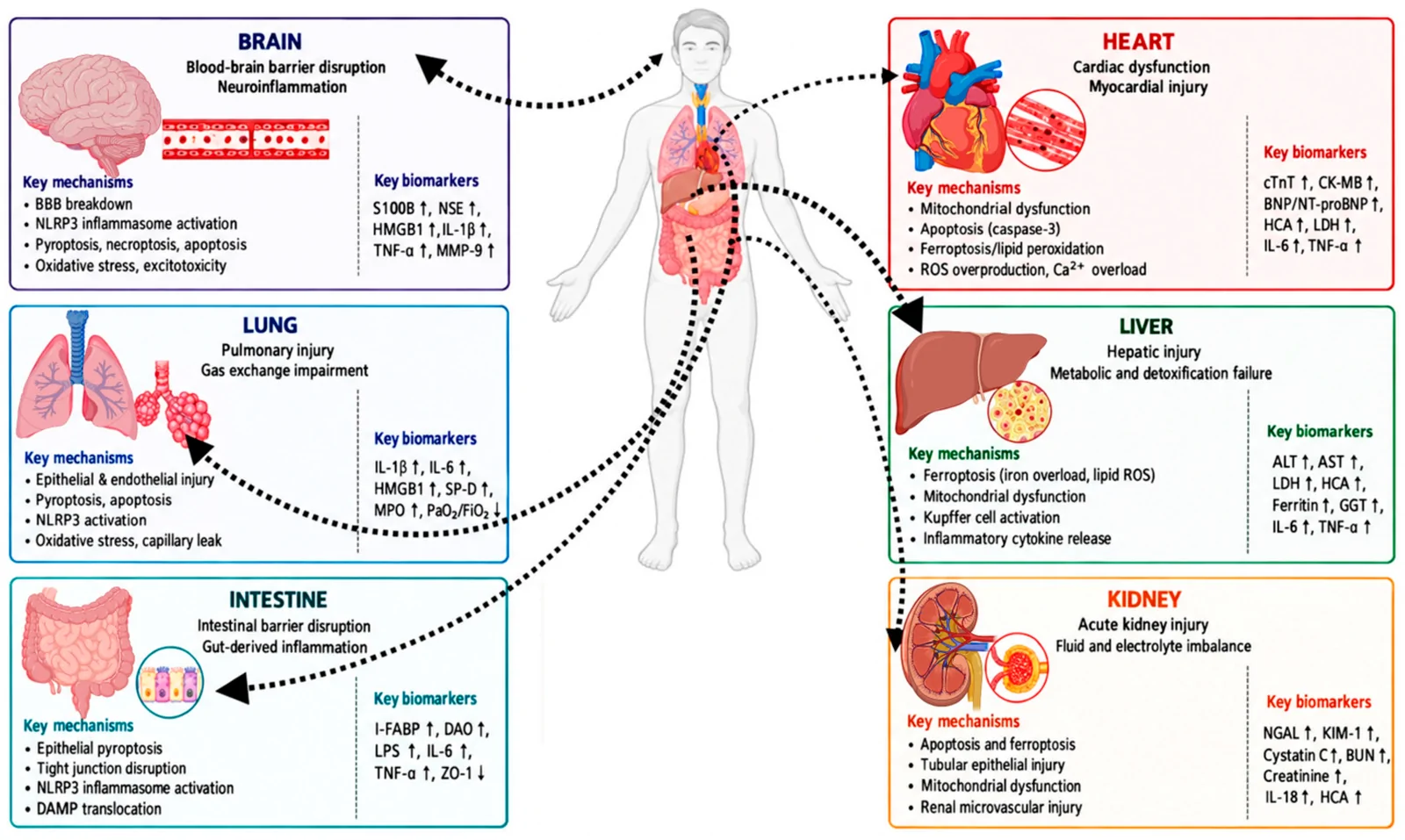
Organ-specific damage pathways that lead to multiorgan dysfunction caused by heat stress. Multiple organ systems are impacted by severe hyperthermia through different but related pathogenic pathways. Disruption of the blood–brain barrier, neuroinflammation, and activation of controlled cell death pathways are characteristics of brain injury. Gas exchange is compromised as a result of oxidative stress, inflammatory signaling, and damage to the epithelium and endothelium. While hepatic injury is linked to metabolic dysfunction, inflammatory cytokine release, and ferroptotic signaling, cardiac dysfunction is caused by mitochondrial damage, oxidative stress, apoptosis, and ferroptosis. Renal injury includes tubular epithelial destruction, mitochondrial dysfunction, apoptosis, and ferroptosis; intestinal barrier breakdown encourages DAMP transfer and systemic inflammation. Created in BioRender. Ajanovic, T. (2026); https://BioRender.com/jrz3c21.

Neurological complications are increasingly recognized as both acute and long-term consequences of thermal injury. Heat-and inflammation-induced disruption of the blood–brain barrier permits infiltration of neutrophils, cytokines, and reactive species into the CNS. At a mechanistic level, cytokines such as TNF-α, IL-1β, and IL-6, along with complement activation (C5a) and oxidative stress, promote microglial activation, neuronal apoptosis, and reduced neurotrophic signaling, contributing to cognitive and functional impairment. Long-term outcomes show a two-fold increase in CNS-related hospitalizations for years following major burns, especially in individuals who sustained injuries before age 45 (Figure 4). Importantly, central nervous system injury in heat-related illness does not occur in isolation but reflects systemic inflammatory and microvascular derangements that simultaneously affect multiple organ systems. These shared mechanisms allow CNS dysfunction to be interpreted as part of a broader, interconnected process of multiorgan failure rather than a standalone endpoint [128,129].

### 6.8. Integrated Model of MODS in Severe Thermal Injury

Across all organ systems, MODS arises from a coherent, escalating cascade:

1. Heat-induced cellular injury → proteostasis collapse and activation of RCD.
2. DAMP release → amplification of innate immune responses and cytokine storm.
3. Endothelial dysfunction → glycocalyx loss and barrier breakdown.
4. Microvascular thrombosis → impaired tissue perfusion and oxygenation.
5. Mitochondrial failure → energetic collapse and oxidative injury.
6. Organ dysfunction affecting cardiovascular, pulmonary, renal, hepatic, intestinal, and neurological systems.
7. System-wide metabolic failure, leading to death in the most severe cases.

In this review, integration was achieved by systematically linking molecular stress responses (proteostasis collapse and oxidative injury) with downstream regulated cell death pathways and further connecting these cellular events to endothelial dysfunction and organ-level failure. Rather than describing pathways in isolation, each section builds a causal sequence from intracellular injury to systemic inflammation and microvascular collapse. This stepwise framework allows cross-talk between apoptosis, ferroptosis, necroptosis, and pyroptosis to be interpreted within a unified pathophysiological continuum. This integrated framework illustrates how diverse molecular and cellular disturbances converge into a single, self-propagating syndrome, shaping both prognosis and therapeutic priorities.

## 7. Biomarkers of Thermal Injury

Biomarkers in heatstroke reflect sequential stages of the underlying pathophysiological cascade rather than isolated diagnostic events. Early markers indicate proteostasis failure, heat shock response activation, and mitochondrial oxidative stress, whereas later markers reflect endothelial injury, glycocalyx degradation, systemic inflammation, coagulopathy, and multiorgan dysfunction. Accordingly, organ-specific biomarkers should be interpreted within the evolving heat stress–RCD–endothelial injury axis. Although biomarkers are valuable for assessing injury severity, progression, prognosis, and forensic relevance, no single marker provides sufficient specificity or sensitivity for diagnosis. Current evidence therefore favors a multimodal panel combining heat shock proteins, inflammatory cytokines, oxidative-stress markers, and mitochondrial DAMPs. However, clinical utility remains limited by incomplete validation, temporal variability, interindividual differences, comorbidities, and methodological heterogeneity. To facilitate interpretation, biomarkers are presented from two complementary perspectives. Table 9 focuses on their biological significance and involvement in the pathophysiological mechanisms of heat-induced injury, whereas Table 10 emphasizes their potential clinical and translational utility for diagnosis, risk stratification, prognosis, and therapeutic monitoring.

Biomarkers may represent several pathophysiological processes, such as proteostasis collapse, oxidative stress, inflammatory amplification, endothelial dysfunction, coagulopathy, and organ failure, given the multistage nature of heat-induced injury. From a translational standpoint, certain biomarkers are already clinically proven, while others are still under investigation or are mainly useful for forensic and experimental purposes. The main biomarkers linked to heat-related damage are compiled in Table 9 based on their translational readiness, clinical usefulness, and biological importance. The clinical and translational classification of biomarkers in heat-related injury are summarized in Table 10.

### 7.1. Clinical Classification and Translational Applicability of Biomarkers

Heat-related biomarkers do not reflect isolated pathological events but rather correspond to sequential stages of injury progression. Early biomarkers are associated with proteostasis collapse and cellular stress responses, including heat shock proteins and markers of mitochondrial dysfunction. Accordingly, the biomarkers discussed below are interpreted not only according to their clinical applicability, but also within the broader mechanistic continuum proposed throughout this review.

From a clinical perspective, biomarkers associated with heat-related injury can be categorized into four major groups: diagnostic biomarkers, severity/prognostic biomarkers, therapeutic monitoring biomarkers, and forensic biomarkers. This classification may be more clinically informative than a purely pathophysiological approach, as it reflects the practical utility of biomarkers in patient management and outcome prediction.

Diagnostic biomarkers are primarily used to support the identification of heatstroke and related tissue injury. Currently, no single biomarker possesses sufficient sensitivity and specificity for standalone diagnosis. Consequently, diagnosis remains largely clinical and is supported by a combination of laboratory abnormalities. Among the most clinically relevant diagnostic biomarkers are IL-6, lactate, D-dimer, platelet count, and markers of endothelial injury such as soluble thrombomodulin and von Willebrand factor (vWF), which reflect systemic inflammation, coagulopathy, and vascular dysfunction associated with severe heat stress [20,59].

Among proteostasis-related biomarkers, heat shock proteins (particularly HSP70 and HSP90) are of special interest because they directly reflect activation and eventual exhaustion of the cellular heat shock response. Increased circulating HSP levels have been associated with severe thermal stress, mitochondrial dysfunction, inflammatory activation, and adverse clinical outcomes. In parallel, biomarkers of proteotoxic injury and mitochondrial damage, including mtDNA and oxidatively modified proteins, may provide insight into the transition from reversible cellular stress toward regulated cell death and systemic inflammation.

Nevertheless, most emerging biomarkers, including HSP70, HMGB1, mitochondrial DNA (mtDNA), and regulated cell death markers, remain investigational and require further validation before routine clinical implementation.

Severity and prognostic biomarkers provide information regarding disease progression, organ dysfunction, and mortality risk. Elevated concentrations of IL-6, lactate, D-dimer, creatinine, troponin, and circulating endothelial injury markers have consistently been associated with increased disease severity, multiorgan dysfunction syndrome (MODS), and poor clinical outcomes. Similarly, thrombocytopenia, prolonged coagulation parameters, and elevated markers of endothelial damage are strongly linked to the development of heatstroke-induced coagulopathy and adverse prognosis [59,146].

Therapeutic monitoring biomarkers are useful for evaluating treatment response and disease evolution. Serial measurements of lactate, IL-6, platelet count, D-dimer, coagulation parameters, creatinine, and liver injury markers (ALT/AST) may assist in assessing the effectiveness of cooling strategies, hemodynamic resuscitation, and organ-supportive therapies. Endothelial injury biomarkers such as syndecan-1 and thrombomodulin may additionally provide insight into ongoing vascular injury and recovery, although their use remains largely confined to research settings. Additional endothelial and glycocalyx-associated biomarkers, including von Willebrand factor (vWF), angiopoietin-2, soluble ICAM-1, VCAM-1, and heparan sulfate fragments, may further reflect endothelial activation, barrier disruption, and microvascular dysfunction during severe heat stress. Because endothelial injury represents a central mechanistic bridge between systemic inflammation, coagulopathy, and organ failure, these biomarkers may have particular value for identifying patients at risk of heatstroke-induced coagulopathy and progressive MODS [59].

Forensic biomarkers have gained increasing attention in the investigation of fatal heat-related injury. Molecules such as HSP70, IL-6, HMGB1, and selected oxidative stress markers may help support postmortem identification of heat exposure and heatstroke-related death. However, interpretation remains challenging because many of these biomarkers are not specific to thermal injury and may also be elevated in sepsis, trauma, or other causes of systemic inflammation [150].

Overall, biomarkers currently used in routine clinical practice, including lactate, D-dimer, platelet count, creatinine, and IL-6, have the highest translational readiness owing to their availability and established prognostic significance. In contrast, HSP70, HMGB1, mtDNA, syndecan-1, ferroptosis-related markers, pyroptosis-associated molecules, and other indicators of regulated cell death remain predominantly exploratory despite their considerable mechanistic relevance. Future studies should focus on standardization, validation, optimal sampling windows, and multimarker strategies capable of integrating inflammation, endothelial dysfunction, coagulopathy, and organ injury into clinically actionable risk assessment models. Within this framework, biomarker interpretation should ideally reflect the temporal and mechanistic progression of heat-induced injury, from early proteostasis disruption and oxidative stress to endothelial dysfunction, coagulopathy, and eventual multiorgan failure.

### 7.2. Heat Shock Proteins (HSPs)

Among all molecular markers studied in the context of thermal trauma, HSP70 has received the most extensive attention. Heat stress rapidly activates HSF1, resulting in a marked transcriptional upregulation of HSP70 that mirrors the degree of intracellular protein unfolding and proteotoxic stress. Elevated levels of HSP70 have been documented in the respiratory tract, lungs, and kidneys of fire victims, where its tissue distribution correlates with survival interval. Serum HSP70 also increases in cases of terminal hyperthermia and correlates with cardiac troponin I, suggesting parallel myocardial and systemic injury. Clinically, higher HSP70 concentrations at admission are associated with larger TBSA involvement and increased in-hospital mortality. Locally, blister-fluid HSP70 rises sharply within hours after injury and then declines as inflammatory processes dominate. However, HSP70 is limited by substantial interindividual variability in both basal and inducible expression, and its levels can be altered by exercise, infection, metabolic conditions, or other stressors. Moreover, once released extracellularly, HSP70 itself can behave as a DAMP, further activating the immune system. Other HSPs, such as HSP27, a small heat shock protein involved in cytoskeletal stabilization, also rise early, including in myocardial and pulmonary tissue, even when survival intervals are very short. Nonetheless, its diagnostic and prognostic utility remains inconsistent due to context-dependent expression and post-translational modifications. HSP90 and HSP60 play important roles in protein homeostasis and stress signaling, but thus far have been studied largely in experimental models, limiting their application in forensic or clinical diagnostics [151,152].

### 7.3. Oxidative Stress Biomarkers

Oxidative stress is a fundamental driver of thermal injury, and several biomarkers reflect this process. Malondialdehyde (MDA), produced during lipid peroxidation, is consistently elevated in burn patients and correlates with TBSA and systemic inflammation. High MDA levels indicate redox imbalance, mitochondrial dysfunction, and impaired antioxidant capacity, although attempts to reduce it with antioxidant therapy have yielded mixed results. 8-Hydroxy-2′-deoxyguanosine (8-OHdG) is a well-established marker of oxidative DNA damage. Levels rise significantly within the first 24 h after severe burns and correlate with TBSA. In animal models, 8-OHdG increases in distant organs and skeletal muscle, and it is likewise elevated in heatstroke due to DNA oxidative injury. However, interpretation is complicated by differences in renal clearance, choice of biological matrix (urine vs. plasma), and the need for high-specificity LC-MS/MS analysis [141,153].

### 7.4. Protein Oxidation and Proteostasis Markers

Markers of protein oxidation provide additional insight into the severity of cellular stress. Protein carbonyls accumulate when ROS attack amino acid side chains, while advanced oxidation protein products (AOPP) increase in early burns as indicators of systemic oxidative load but do not distinguish burn depth. Another potential marker of proteostasis failure is the accumulation of ubiquitinated proteins, reflecting saturation of proteasomal degradation pathways under severe heat stress. Although informative, these markers lack standardized assays suitable for routine clinical use [154].

### 7.5. Mitochondrial Damage-Associated Molecular Patterns (mtDAMPs)

Mitochondrial dysfunction during heat injury leads to the release of circulating mitochondrial DNA (mtDNA), one of the most potent triggers of sterile inflammation. mtDNA’s unmethylated CpG motifs activate TLR9, the NLRP3 inflammasome, and the cGAS–STING pathway, driving strong inflammatory and endothelial-activating responses. Elevated mtDNA correlates with systemic inflammation, microvascular injury, and adverse outcomes in critical illness, making it a promising biomarker of severity in both heatstroke and major thermal trauma. Its relevance lies in coupling three pathological domains, mitochondrial injury, DAMP signaling, and coagulopathy, that are central to the heatstroke cascade [155].

### 7.6. Postmortem Biomarkers

In forensic contexts, biomarker research has identified several liver-derived proteins that may help distinguish heatstroke-related deaths. These include HSPA4, a member of the HSP70 family reflecting depletion of CSR; GGCX, which connects hepatic dysfunction to coagulopathy; and CYP2B6, a marker of hepatocellular metabolic collapse. Additionally, ATR-FTIR spectroscopy has shown promise in differentiating fatal hyperthermia from hypothermia and non-thermal deaths by assessing lipid, carbohydrate, and nucleic-acid spectral patterns, although hemolysis and postmortem changes may affect accuracy [155,156]. In addition, the evaluation of histological, immunohistochemical, and molecular indicators of vital reactions in thermal injury has been reviewed as a complementary approach to forensic interpretation [157].

### 7.7. Limitations and Future Directions

Across all biomarker categories, several recurring limitations persist. Many lack specificity for heat injury, as they also rise in infection, trauma, ischemia, or systemic inflammation. Baseline levels vary widely due to genetics, comorbidities, age, sex, hydration status, and lifestyle factors. Most biomarkers exhibit dynamic fluctuations, necessitating standardized sampling protocols that remain inconsistently applied. There is also a broader need for multicenter validation, especially for promising oxidative and mitochondrial markers, and for biomarkers that can reliably support forensic determinations of vitality.

Future research should emphasize the following:

- The development of integrated multimarker panels combining HSPs, cytokines, oxidative stress markers, and mtDNA;
- Longitudinal sampling to track biomarker kinetics across the hyperacute, acute, and recovery phases;
- The incorporation of multi-omics approaches (transcriptomics, proteomics, metabolomics) to uncover composite molecular signatures;
- Validation in large, diverse patient populations to support both clinical and forensic application.

## 8. Therapeutic Perspectives: From Antioxidants to Endothelial Protection

Current therapeutic strategies for heat- and burn-induced systemic injury remain limited, and no targeted pharmacologic therapy is yet standard of care. Management relies largely on rapid cooling, hemodynamic stabilization, and organ support, while molecularly directed interventions are still emerging. Despite the growing interest in mechanism-based therapeutic strategies, it is important to emphasize that rapid cooling remains the cornerstone of heatstroke management and the only intervention currently supported by consistent clinical evidence. Early reduction in core body temperature is strongly associated with improved survival and reduced organ injury and therefore remains the primary therapeutic objective in both exertional and non-exertional heat stroke. Consequently, the molecular targets discussed in this review should be viewed as potential adjunctive or future therapeutic approaches rather than alternatives to prompt cooling and supportive care. A mechanistic understanding of proteostasis collapse, RCD, endothelial dysfunction, oxidative stress, and immune activation provides a rationale for novel therapies aimed at interrupting the cascade that leads to HIC and MODS [60,156].

### 8.1. Modulation of Heat Shock Proteins (HSPs)

Given their role in cellular stabilization, HSPs, especially Hsp90, HSP70, and HSP27, represent promising therapeutic targets. Experimental work demonstrates that Hsp90 activation of the PKM2–Akt axis suppresses apoptosis and enhances HSP70 induction via HSF-1 activation, thereby improving heat tolerance and limiting histopathological injury. Hsp90 also augments autophagy-mediated cytoprotection through the HIF-1α–BNIP3/BNIP3L pathway, suggesting a dual role in coordinating proteostasis and mitochondrial quality control. In endothelial cells, HSP27 supports NF-κB activation, limits ROS accumulation, and reduces apoptosis; knockdown reverses these protective effects. Although these findings highlight promising cytoprotective mechanisms, HSP-targeted therapies have not yet translated to clinical practice [158,159,160].

### 8.2. Antioxidant-Based Interventions

Because oxidative stress is a major driver of endothelial injury, inflammatory amplification, and coagulopathy in heat-related illness, numerous therapeutic efforts have focused on antioxidant strategies. Vitamin C (ascorbic acid) has been studied most extensively. In experimental burn plasma models, high-dose vitamin C is able to substantially reduce endothelial hyperpermeability, restoring barrier function to near-baseline levels, although it does not significantly diminish leukocyte adhesion. Clinically, observational data suggest that administering more than 10 g within the first 48 h may reduce mortality, but these findings remain inconsistent and highlight the need for larger, well-controlled trials. Another promising agent is melatonin, which acts as a potent scavenger of reactive oxygen and nitrogen species. In burn models, it enhances endogenous antioxidant defenses, limits distant organ injury, and reduces immune dysregulation. Despite these encouraging preclinical results, melatonin has not yet been validated in large human cohorts. Overall, the therapeutic impact of antioxidants remains mixed, likely because oxidative injury in heatstroke is multifactorial and highly time-dependent, making the timing and context of administration critical [161,162,163].

### 8.3. Targeted Modulation of Inflammatory Pathways

Given the central role of dysregulated inflammation in heat injury, several interventions target specific cytokines and signaling pathways. Local inhibition of TNF-α, a major upstream inflammatory mediator, has been shown to reduce burn wound progression, limit neutrophil recruitment, and decrease secondary necrosis, performing better than IL-6 blockade in preclinical studies. However, despite strong mechanistic rationale, cytokine-targeted biologics have not yet translated into consistent clinical benefit for patients with heatstroke or severe burns. Other approaches, such as adenosine A_2_A receptor agonists, reduce neutrophil infiltration, microvascular thrombosis, and burn depth progression in porcine models. Their combined anti-inflammatory and endothelial-protective effects make them attractive candidates for early intervention. Similarly, inhibition of the p38 MAPK pathway has been shown to reduce DAMP release and attenuate remote lung inflammation, suggesting a role in preventing the systemic propagation of inflammatory signals. Therapies targeting extracellular ATP, such as apyrase, can limit neutrophil infiltration and slow necrotic expansion in partial-thickness burns. Together, these findings indicate that early, localized modulation of inflammation offers substantial therapeutic promise, even though robust human studies remain lacking [163,164].

The limited success of anti-inflammatory therapies in clinical settings may reflect the complexity and redundancy of the underlying signaling pathways, as well as the fact that inflammation often represents a secondary amplification stage rather than the primary initiating event. This may partly explain why strategies targeting downstream cytokines have shown inconsistent clinical benefit, suggesting that upstream processes, such as proteostasis disruption and early endothelial injury, may represent more effective therapeutic targets.

### 8.4. Endothelial Protection and Coagulopathy Modulation

Because endothelial injury is a central hallmark of heatstroke and burn-related systemic illness, therapies aimed at preserving the glycocalyx, stabilizing intercellular junctions, and modulating coagulation may have the greatest translational potential. Agents such as dexmedetomidine demonstrate strong glycocalyx-protective effects, reducing vascular leakage and improving survival in heat-induced injury models. Antioxidants such as N-acetylcysteine (NAC) can further support endothelial health by attenuating glycocalyx shedding and improving coagulation parameters. A number of coagulation-modifying therapies also show promise. Activated protein C improves survival and mitigates endothelial injury in heatstroke models. Recombinant thrombomodulin suppresses HMGB1 release, restores protein C activity, and yields significant survival benefits. Additionally, sodium tanshinone IIA sulfonate reduces DIC (DIC) and organ damage in rat models. Collectively, these interventions act at the critical intersection where inflammation, endothelial disruption, and microthrombosis converge, aligning closely with the pathophysiology of HIC [26,94].

### 8.5. Therapies Targeting Mitochondrial Dysfunction

Mitochondrial failure is a defining feature of severe heat injury, contributing to ROS overload, ATP depletion, and activation of regulated cell death pathways. In this context, mitophagy, particularly the PINK1/Parkin pathway, plays a central role in clearing damaged mitochondria and maintaining cellular homeostasis. Enhancing this pathway, either pharmacologically or through USP30 inhibition, has been shown to reduce mitochondrial injury and promote cellular recovery in experimental systems. The mitochondrial deacetylase Sirt3 may further support PINK1/Parkin-dependent mitophagy and facilitate tissue repair. Despite the mechanistic appeal of these strategies, no mitophagy-targeted therapy has yet advanced to clinical readiness [6,76].

### 8.6. Nanomedicine-Enhanced Wound and Tissue Repair

Nanotechnology offers a versatile platform for treating burn and heat injuries, with advantages that include enhanced antioxidant capacity, antimicrobial action, improved angiogenesis, and controlled drug release. Several classes of nanoparticles have shown promise: silver nanoparticles (Ag NPs) for their broad antimicrobial activity and ability to improve re-epithelialization; zinc oxide nanoparticles (ZnO NPs) for stimulating keratinocyte proliferation; gold nanoparticles (Au NPs) for their anti-inflammatory and antioxidative properties; and polymer-based nanofibers such as chitosan, collagen, and PLGA, which provide extracellular matrix-mimicking scaffolds that support tissue regeneration. Plant-derived compounds such as asiaticoside, madecassoside, and asiatic acid, extracted from Centella asiatica, add further potential through their antioxidant, collagen-stimulating, and pro-angiogenic effects, which accelerate wound healing. Despite these promising results, nanotherapies remain in the early experimental stage, and human safety and efficacy data are still limited [165,166].

### 8.7. Integrative Perspective: Combining Proteostasis, Inflammation, and Endothelial Targets

Heat injury arises from an intricate interplay between proteostasis collapse, oxidative stress, inflammatory activation, and endothelial dysfunction, suggesting that single-target therapies are unlikely to produce adequate clinical benefit. As summarized in Table 11, effective treatment will instead require multi-target strategies that address several pathological axes simultaneously, for example, combining HSP-enhancing agents with antioxidants, anti-inflammatory therapies, and endothelial-protective interventions. Approaches that simultaneously preserve the glycocalyx, restore proteostasis, and prevent microvascular thrombosis may hold the greatest promise. Ultimately, future therapeutic regimens will likely be guided by patient-specific biomarker profiles, supported by real-time diagnostics that track oxidative stress, immune activation, mitochondrial injury, and endothelial integrity. Despite these promising mechanistic advances, the majority of targeted therapeutic approaches discussed in this review remain supported primarily by preclinical or early translational evidence. Apart from established supportive measures and rapid cooling strategies, no mechanism-based intervention has yet demonstrated sufficient clinical efficacy to warrant routine recommendation in heatstroke patients [101,102,105]. Nevertheless, regardless of future advances in targeted therapies, rapid recognition of heat stroke and immediate cooling remain the fundamental determinants of patient outcome and continue to represent the current standard of care. Therefore, these approaches should currently be considered investigational rather than evidence-based clinical therapies. To facilitate interpretation, Table 11 summarizes the current evidence level, principal biological effects, and major translational limitations associated with each therapeutic strategy.

Given its central position at the interface between inflammation, coagulation, and microvascular function, endothelial protection may represent the most promising translational target within the proposed framework.

Although the therapeutic approaches discussed above demonstrate considerable promise, substantial challenges remain regarding patient stratification, biomarker validation, treatment personalization, predictive modeling, and mechanistic integration. These broader scientific and translational challenges are discussed in the following section, which focuses on future research directions and emerging precision-medicine frameworks.

## 9. Future Directions and Research Priorities

Future advances in the understanding and treatment of heat-related injury will depend on a deeply integrated research strategy that bridges molecular biology, systems immunology, high-resolution omics, computational science, precision medicine, and biomaterials engineering. Although substantial progress has been made in delineating the mechanisms underlying proteostasis collapse, RCD, endothelial dysfunction, and systemic inflammation, significant challenges remain in translating these mechanistic insights into validated biomarkers, targeted therapeutics, and practical clinical tools.

### 9.1. Single-Cell and Spatial Omics to Resolve Tissue Heterogeneity

Traditional bulk tissue analyses have long been limited by their inability to capture the complexity and heterogeneity of cellular responses during heat injury. Emerging single-cell multi-omics approaches, including scRNA-seq, CITE-seq, REAP-seq, ASAP-seq, and NEAT-seq, now allow researchers to profile transcriptomes, proteomes, and chromatin accessibility at the resolution of individual cells. These methods can distinguish between injured keratinocytes, activated endothelial cells, neutrophil and macrophage subsets, and heat-stressed epithelial barriers, uncovering regulatory programs that are invisible in mixed-cell analyses. Spatial transcriptomics expands this capability by preserving tissue architecture, enabling researchers to map inflammatory mediators, glycocalyx injury markers, mitochondrial stress signatures, and transcriptional regulators such as NF-κB and SIRT1 within their precise anatomical context. These tools hold particular promise for forensic pathology, allowing for differentiation between antemortem and postmortem heat injury based on localized molecular signatures. Despite the clear potential, these technologies are limited by high costs, complex sample preparation, and the need for sophisticated computational analysis; nevertheless, they are poised to shape the next generation of diagnostic and investigative approaches [166,167].

### 9.2. Integrating Multi-Omics and Machine Learning for Predictive Modeling

Machine learning approaches may facilitate the integration of complex clinical and biomarker data in heat-related injury. Future studies should focus on validating predictive models that incorporate dynamic biomarkers, including HSPs, circulating mtDNA, cytokines, and coagulation markers, to improve risk stratification and prognostic assessment. However, broader clinical implementation will require large multicenter datasets, external validation, and improved model interpretability [168,169].

### 9.3. Personalized Medicine and Genetic Susceptibility

Growing evidence suggests that susceptibility to heat stress varies substantially among individuals and may be influenced by genetic polymorphisms in heat shock protein genes such as HSPA1B, HSP70 family members, HSP90, and regulatory elements like HSF1. Although human studies remain limited, identifying such variants could help stratify risk among athletes, military personnel, and outdoor workers, and could also inform personalized cooling strategies, acclimatization programs, and individualized therapeutic interventions, such as targeted modulation of HSP expression. Incorporating genetic data alongside measured biomarker responses, environmental exposures, and computational predictions will ultimately support precision-medicine approaches tailored to each individual’s heat injury risk profile [170,171,172].

### 9.4. Advancing Nanotechnology-Driven Therapies

From a future research perspective, nanomedicine also offers an important experimental platform for investigating tissue-specific modulation of proteostasis, inflammatory signaling, and endothelial protection mechanisms. Nanoparticle platforms offer targeted drug delivery, enhanced oxidative stress mitigation, modulation of inflammatory pathways, antimicrobial activity, and endothelial protection. Future research may employ nanoparticles to deliver antioxidants, HSP modulators, or endothelial stabilizers, while nanofiber scaffolds that mimic the extracellular matrix may promote improved wound healing. Additional innovations include magnetic or photothermal nanoparticles for controlled hyperthermia modulation and nano-antibiotic formulations capable of overcoming antibiotic resistance. Yet for these technologies to progress toward clinical use, challenges such as biocompatibility, biodistribution, long-term safety, and regulatory approval must be addressed through rigorous preclinical and clinical evaluation [173,174,175,176,177,178].

### 9.5. Improving Biomarker Panels and Diagnostic Algorithms

Unlike the therapeutic applications discussed in Section 8, the emphasis here is placed on biomarker-guided patient stratification, prognostic modeling, and precision-medicine approaches capable of identifying biologically distinct heatstroke phenotypes. Biomarker research in thermal injury remains fragmented, with substantial variability in sampling times, assay methods, and cohort demographics, which complicates interpretation. To improve diagnostic accuracy and prognostic utility, future research should focus on standardizing protocols for measuring HSPs, cytokines, oxidative stress markers, and mtDNA across centers. There is also a need to develop multiplexed biomarker panels that combine complementary indicators of proteostasis collapse, inflammation, endothelial dysfunction, and mitochondrial stress. Validation of such panels in large, multi-ethnic, multicenter studies will be essential. Moreover, integrating single-cell and spatial-omics signatures into diagnostic algorithms and developing point-of-care assays, such as rapid mtDNA quantification or HSP70 lateral-flow tests, could significantly enhance both clinical management and forensic diagnostic accuracy in heat-related deaths.

### 9.6. Refining Computational and Thermodynamic Models

Existing computational models often oversimplify biological complexity and lack robust clinical validation. Future modeling efforts should strive to create multiscale frameworks that connect molecular processes, such as RCD and endothelial injury, to organ-level perfusion, microvascular dynamics, and systemic physiological changes. By incorporating patient-specific variables such as age, comorbidities, genetic predispositions, and hydration status, these models could provide individualized predictions of injury progression. Simulations exploring glycocalyx damage kinetics, microvascular thrombosis, and optimal cooling thresholds may provide new insights into early intervention strategies and guide real-time decision support in prehospital and emergency settings [174,175].

### 9.7. Translational and Regenerative Strategies

Regenerative medicine is poised to play an expanding role in the treatment of heatstroke and burn injuries. Key priorities include therapies that enhance mitochondrial quality control, such as PINK1/Parkin activation or Sirt3 modulation, which may improve cellular resilience under thermal stress. Strategies that augment autophagy and proteostasis could help prevent collapse of intracellular defenses during severe hyperthermia. Additional approaches involve developing agents that protect the endothelial glycocalyx, exploring stem-cell-derived exosomes to deliver regenerative and anti-inflammatory mediators, and designing bioengineered skin substitutes optimized for thermal-injury repair. Bridging the gap between mechanistic insights and clinically applicable therapies will be essential to advancing these promising modalities [176,177,178,179].

### 9.8. Toward a Unified Research Framework

Progress in the field will depend on coordinated research efforts and integrated infrastructures. Establishing international registries of heatstroke and thermal injury, alongside standardized diagnostic and biomarker protocols, would greatly facilitate cross-study comparisons. Effective integration of omics data, advanced imaging, computational models, and clinical information will enable a far more comprehensive understanding of thermal pathophysiology. Collaboration across disciplines, spanning burn surgeons, critical-care specialists, molecular biologists, bioengineers, and data scientists, will be essential. Finally, the creation of open-access datasets will promote transparency, reproducibility, and scientific acceleration. Together, these efforts will critically enhance discovery, improve patient outcomes, and strengthen forensic reliability [180,181,182,183].

## 10. Conclusions

Heat-induced injury represents a complex and multilayered biological process in which proteostasis collapse, mitochondrial dysfunction, RCD, DAMP-driven inflammation, endothelial disruption, and coagulopathy interact to generate rapidly progressive systemic damage. This review has brought these elements together into a unified mechanistic framework, illustrating how an initial disturbance in protein homeostasis under thermal stress can cascade into vascular barrier failure, microthrombotic injury, and ultimately MODS.

Viewed across molecular, cellular, and organ-system scales, a consistent theme emerges: heatstroke and severe burns should not be regarded merely as thermoregulatory disorders, but rather as systemic endothelial and immunometabolic syndromes. The progression from early heat shock response activation to chaperone saturation, mitochondrial collapse, and engagement of multiple regulated cell death pathways provides the biological substrate for explosive DAMP release, cytokine storm generation, and the shift to a profoundly pro-coagulant endothelial phenotype. Together, these intertwined mechanisms drive HIC and ultimately MODS, the primary determinant of clinical outcomes in critical heat injury.

Despite considerable advances in mechanistic understanding, important gaps remain. Current biomarkers lack sufficient specificity and reliability, underscoring the need for integrated, multimarker approaches. Many promising therapeutic avenues, from HSP-modulating agents and antioxidants to endothelial-protective strategies, anti-inflammatory interventions, mitophagy enhancers, and nanomedicine platforms, remain largely preclinical or insufficiently validated in human studies. Meanwhile, emerging innovations such as high-resolution omics, machine learning models, and computational simulations offer transformative potential but face barriers related to technical complexity, cost, and standardization. Notably, several fundamental questions remain unresolved, including the relative contribution of upstream cellular stress versus downstream inflammatory amplification, the organ-specific dominance of different regulated cell death pathways, and the continuing gap between strong preclinical evidence and limited clinical translation of targeted therapies.

Looking ahead, meaningful progress will depend on cross-disciplinary integration: combining multi-omics profiling, endothelial biology, computational prediction, and targeted pharmacology within harmonized clinical and forensic frameworks. The creation of large-scale, multicenter datasets and standardized biomarker protocols will be essential for improving diagnostic precision, enabling personalized risk stratification, and supporting rational therapeutic development. Concurrently, advances in regenerative medicine and nanotechnology are poised to reshape future approaches to organ protection and wound repair.

An additional challenge concerns the interpretation of mechanistic evidence derived from heterogeneous experimental systems. Although this review integrates findings from heatstroke, burn injury, hyperthermia, and related inflammatory models, these conditions are not pathophysiologically identical. Therefore, some mechanistic connections proposed herein should be regarded as hypothesis-generating extrapolations rather than heatstroke-specific conclusions, underscoring the need for dedicated validation in human heatstroke and experimental heatstroke models.

Although substantial evidence supports the individual molecular components discussed throughout this review, including proteostasis disruption, oxidative stress, mitochondrial dysfunction, regulated cell death, DAMP signaling, endothelial injury, and coagulopathy, direct experimental proof that these events are organized by a single proteostasis-centered upstream mechanism remains incomplete. Accordingly, the proposed framework should be viewed as a hypothesis-driven integrative model that synthesizes available findings across multiple experimental and clinical domains rather than as a definitively established causal pathway. By integrating mechanistic depth with translational relevance, future research can accelerate the development of targeted interventions and improve outcomes for individuals exposed to life-threatening thermal stress. To avoid being a fully validated causal pathway, the framework presented in this review should be understood as a hypothesis-driven integrative model based on current experimental and clinical findings. To determine the temporal and causal links between proteostasis failure, regulated cell death, inflammation, endothelial dysfunction, and coagulopathy in heatstroke, more mechanistic and translational research is needed.

This review proposes an integrative molecular-to-systemic framework linking intracellular proteostasis disruption to endothelial injury, coagulopathy, multiorgan dysfunction, and death. This framework should be regarded as a hypothesis-driven model that may guide future mechanistic and translational research in heatstroke.

## Figures and Tables

**Table 1:** A comparative summary of the characteristics of major heat stress-associated cell death pathways.

Cell Death Pathway	Main Trigger	Key Molecular Mediators	Main Characteristics	Inflammatory Potential	Representative Biomarkers	References
Apoptosis	Moderate heat stress, mitochondrial injury	Caspase-9, caspase-3, BAX/BAK, cytochrome c	Controlled ATP-dependent cell dismantling	Generally low but context-dependent	Caspase-3 activation, Bax/Bcl-2 ratio, cytochrome c	[12,13,14]
Pyroptosis	Inflammasome activation, DAMP signaling	NLRP3, caspase-1, GSDMD	Membrane pore formation and cytokine release	Often high, context-dependent	IL-1β, IL-18, cleaved GSDMD	[39,42,43]
Ferroptosis	Oxidative stress, iron overload	GPX4, lipid ROS, glutathione depletion	Iron-dependent lipid peroxidation	Variable, context-dependent	GPX4 downregulation, lipid ROS	[48,49,50]
Necroptosis	Caspase-8 inhibition, TNF signaling	RIPK1, RIPK3, MLKL	Programmed membrane rupture	Often high, context-dependent	Phosphorylated MLKL, RIPK3	[45]
Necrosis	Extreme thermal injury, ATP depletion	Non-regulated cellular collapse	Rapid membrane destruction	Extreme	Massive DAMP release, membrane rupture	[46,50]

**Table 2:** Therapeutic strategies in heatstroke categorized according to regulated cell death (RCD) pathways and mechanistic targets.

Therapeutic Strategy	Targeted RCD Pathway/Biological Process	Key Mechanistic Targets	Main Therapeutic Effect	References
HSP modulation/chaperone induction	Proteostasis failure → Apoptosis (early stress response)	HSF1 activation, HSP70/HSP90 induction, protein folding restoration	Stabilization of proteome and reduction in early apoptotic signaling	[54]
Antioxidant therapy (e.g., NAC)	Apoptosis, Necroptosis, upstream oxidative stress	ROS scavenging, mitochondrial protection, glutathione replenishment	Reduction in oxidative damage and suppression of RCD initiation	[28]
Ferroptosis inhibitors	Ferroptosis	GPX4 preservation, lipid ROS inhibition, iron chelation	Prevention of iron-dependent lipid peroxidation and membrane injury	[48]
NLRP3 inflammasome inhibitors	Pyroptosis	NLRP3 suppression, caspase-1 inhibition, IL-1β/IL-18 reduction	Attenuation of inflammatory lytic cell death	[55]
Necroptosis inhibitors (e.g., necrostatin-1)	Necroptosis	RIPK1/RIPK3/MLKL pathway inhibition	Reduction in membrane rupture, endothelial injury, and thrombosis	[48]
Apoptosis modulators	Intrinsic/extrinsic apoptosis	BCL-2 family regulation, caspase-8/9/3 modulation	Control of early regulated cell dismantling	[49]
Mitophagy enhancers	Mitochondrial dysfunction/PANoptotic convergence	Clearance of damaged mitochondria, reduction in mtROS	Limitation of oxidative stress and prevention of pathway convergence	[56]
Endothelial-protective therapies	Endothelial dysfunction/coagulopathy	Glycocalyx stabilization, barrier integrity, anti-thrombotic effects	Prevention of vascular leakage and microvascular thrombosis	[57]
Nanomedicine-based targeted delivery	Multi-pathway RCD modulation	Targeted delivery of antioxidants/inhibitors to injured tissues	Increased specificity and reduced systemic toxicity	[58]

**Table 4:** Pattern Recognition Receptors (PRRs) Involved in Heat Injury.

Receptor	Key Ligands in Heat Injury	Downstream Signaling	Primary Cytokines Produced	Role in Heat Injury	References
TLR2	HMGB1; ECM fragments	MyD88 → NF-κB	IL-1β; TNF-α; IL-6	Major driver of burn-related inflammation	[70]
TLR4	HMGB1; S100 proteins; histones	MyD88/TRIF → NF-κB, IRF	IL-1β; IL-6; IL-8; TNF-α	Amplifies cytokine storm; promotes endothelial activation	[71]
NLRP3	mtDNA; ATP; ROS; K^+^ efflux	Inflammasome → Caspase-1	IL-1β; IL-18	Initiates pyroptosis; systemic hyperinflammation	[72,73]
RAGE (AGER)	HMGB1; S100s; ECM breakdown products	NF-κB amplification loop	IL-6; TNF-α; chemokines	Critical in endothelial injury and late inflammation	[68,74]
NLRP1	Minimal involvement in heat injury	—	—	Likely negligible role	[75,76]

**Table 5:** Evidence strength for DAMP/PRR/inflammasome pathways in heat-related injury. Evidence grading was based on the consistency of available findings, study design, and degree of mechanistic validation. “Strong” indicates convergent evidence from multiple experimental and/or clinical studies with substantial mechanistic support; “Moderate” indicates evidence supported by several studies but with incomplete mechanistic confirmation; “Limited” indicates preliminary, indirect, or emerging evidence. Experimental studies were weighted primarily for assessment of causal and mechanistic relationships, whereas clinical observational studies were primarily considered evidence of biological or clinical association.

Pathway/Mediator	Function	Evidence Strength	References
HMGB1–TLR4/RAGE	DAMP-driven inflammation	Moderate	[26,68,92]
mtDNA–TLR9/cGAS-STING/NLRP3	Mitochondrial danger signaling	Moderate	[93,94]
ATP–P2X7–NLRP3	Inflammasome activation	Moderate	[95,96]
Histones/nuclear DAMPs	Endothelial injury and coagulopathy	Limited	[97,98]
S100 proteins–RAGE/TLR4	Inflammatory amplification	Limited–moderate	[99,100]
NLRP3 inflammasome	Pyroptosis and cytokine maturation	Strong	[72]
IL-1β/IL-18	Pro-inflammatory cytokines	Moderate–strong	[81,101,102]
IL-6/TNF-α/IL-8	Cytokine storm mediators	Strong	[103]

**Table 6:** Mechanisms of Endothelial Injury During Hyperthermia.

Mechanism	Trigger(s)	Structural Effect	Functional Consequence	Relevant Biomarkers	References
Glycocalyx degradation	ROS; proteases; TNF-α; IL-1β	Syndecan-1 and hyaluronan loss	Increased permeability; leukocyte adhesion	Syndecan-1; HA	[50,101]
Junctional destabilization	MLCK activation; Ca^2+^ overload; cytokines	Claudin-5, VE-cadherin disruption	Paracellular leak; edema	Claudin-5 loss; TEER ↓	[102,106]
Oxidative stress	Mitochondrial ROS; NOX activation	Membrane lipid oxidation	Reduced NO; vasoconstriction; microvascular collapse	MDA; 8-OHdG	[50,106]
Pro-coagulant switch	TF expression; vWF release	Endothelial activation	Microthrombosis; DIC	TF; vWF	[50,107]

**Table 7:** Distinguishing Features of Heatstroke-Induced Coagulopathy (HIC) and Conventional Disseminated Intravascular Coagulation (DIC).

Feature	Heatstroke-Induced Coagulopathy (HIC)	Conventional DIC	References
Primary trigger	Extreme hyperthermia and direct thermal tissue injury	Infection, trauma, malignancy, obstetric complications	[59,110,111]
Initiating signals	Predominantly DAMPs (HMGB1, extracellular histones, mtDNA, ATP)	Predominantly PAMPs and inflammatory mediators	[59,110]
Endothelial injury	Early, direct, and central pathogenic event	Secondary to systemic inflammatory activation	[6,59]
Inflammatory milieu	Sterile inflammation with cytokine storm (IL-1β, IL-6, TNF-α)	Pathogen-driven inflammatory response	[59,111]
Coagulation activation	Endothelial dysfunction, tissue factor expression, platelet activation, immunothrombosis	Systemic tissue factor-mediated coagulation activation	[4,59,111]
Fibrinogen	Initially normal/elevated; decreases in advanced stages	Often decreased due to consumption	[4,59,111]
D-dimer/FDPs	Elevated	Elevated	[4,59]
Protein C pathway	Reduced anticoagulant activity	Reduced activity and consumption	[59,111]
Dominant mechanism	Endotheliopathy-driven thromboinflammation	Consumptive coagulopathy	[4,59,111]
Clinical outcome	Heatstroke-associated MODS and organ failure	Organ dysfunction, thrombosis and bleeding	[4,59]

**Table 8:** Organ-Specific Mechanisms of MODS in Heat Injury.

Organ/System	Primary Mechanism(s)	Key Mediators	Clinical Consequences	References
Heart	Mitochondrial dysfunction; cytokine-mediated depression; β-adrenergic exhaustion	TNF-α; IL-1β; ROS; catecholamines	Reduced EF; arrhythmias; shock	[113]
Lungs	Airway epithelial destruction; alveolar inflammation; surfactant dysfunction	ROS; neutrophils; toxins (CO, HCN)	ARDS; airway obstruction	[114]
Kidneys	Hypoperfusion; rhabdomyolysis; tubular oxidative injury	Myoglobin; hemoglobin; RAAS	AKI; acidosis; high mortality	[115]
Liver	Acute-phase dysregulation; steatosis	IL-6; TNF-α	Coagulopathy; metabolic failure	[116]
Intestine	Ischemia; barrier breakdown	LPS; DAMPs	Endotoxemia; SIRS	[117]
Brain	BBB disruption; microglial activation	TNF-α; IL-1β; ROS; C5a	Edema; cognitive impairment; seizures	[118]

**Table 9:** Pathophysiological biomarkers associated with thermal injury and heatstroke.

Pathophysiological Stage	Representative Biomarkers	Biological Significance	Potential Clinical Relevance	References
Proteostasis collapse/heat shock response	HSP70, HSP90	Cellular stress response; chaperone activation	Early heat stress detection; severity assessment	[128]
Oxidative stress and mitochondrial dysfunction	ROS, MDA, 8-OHdG, mtDNA	Lipid peroxidation, oxidative DNA damage, mitochondrial injury	Assessment of oxidative burden and tissue injury	[129]
Apoptosis	Caspase-3, cytochrome c	Mitochondrial apoptotic signaling	Cell death monitoring	[130]
Pyroptosis/inflammasome activation	NLRP3, IL-1β, IL-18, GSDMD	Inflammatory cell death and cytokine activation	Hyperinflammatory response assessment	[131]
Ferroptosis	GPX4 depletion, lipid ROS, ferritin	Iron-dependent lipid peroxidation	Oxidative membrane injury evaluation	[81]
Necroptosis	RIPK1, RIPK3, MLKL	Programmed necrotic signaling	Severe inflammatory tissue injury	[132]
DAMP release and innate immune activation	HMGB1, ATP, S100 proteins	Sterile inflammation and PRR activation	Systemic inflammatory progression	[133]
Cytokine storm	IL-6, TNF-α, IL-8	Hypercytokinemia and immune dysregulation	Severity and mortality prediction	[134]

**Table 10:** Diagnostic, prognostic and translational applications of biomarkers in thermal injury and heatstroke.

Biomarker	Main Pathophysiological Process	Biological Matrix	Potential Clinical Application	Prognostic Value	Translational Readiness	References
HSP70	Proteostasis collapse/cellular stress	Serum, tissue	Early heat stress detection	Moderate	Exploratory-to-translational	[103]
IL-6	Cytokine storm/systemic inflammation	Serum, plasma	Severity assessment; inflammatory monitoring	High	Clinically relevant	[135]
IL-8	Neutrophil recruitment/inflammation	Serum, plasma	Hyperinflammatory response assessment	Moderate	Translational	[136]
TNF-α	Early inflammatory activation	Serum, plasma	Inflammatory activity monitoring	Moderate	Translational	[137]
MDA	Lipid peroxidation/oxidative stress	Serum, tissue	Oxidative injury assessment	Moderate	Exploratory	[138]
8-OHdG	Oxidative DNA damage	Urine, serum	Oxidative stress monitoring	Moderate	Exploratory	[139]
mtDNA	Mitochondrial injury/DAMP release	Plasma, serum	Systemic injury and inflammatory activation	Potentially high	Experimental	[140]
HMGB1	Sterile inflammation/DAMP signaling	Serum, plasma	Endothelial and inflammatory injury assessment	High potential	Experimental-to-translational	[141]
Syndecan-1	Glycocalyx degradation	Plasma	Endothelial injury monitoring	High	Translational	[142]
Thrombomodulin	Endothelial dysfunction/coagulopathy	Plasma	HIC severity assessment	High	Translational	[143,144,145]
D-dimer	Coagulation activation/thrombosis	Plasma	Coagulopathy monitoring	High	Clinically established	[146]
vWF	Endothelial activation	Plasma	Microvascular injury assessment	Moderate-to-high	Translational	[147]
Lactate	Tissue hypoperfusion/MODS	Blood	Shock and mortality assessment	High	Clinically established	[148]
Creatinine	Renal injury/MODS	Serum	AKI monitoring	High	Clinically established	[149]
HSP70, IL-6, HMGB1 (postmortem)	Heat exposure/systemic inflammation	Postmortem tissue, blood	Forensic interpretation	Moderate	Exploratory forensic application	[150]

**Table 11:** Therapeutic Strategies Mapped to Mechanistic Targets.

Therapeutic Target	Representative Approaches	Main Benefit	Development Stage	References
Proteostasis	HSP modulators	Cytoprotection	Preclinical	[153]
Oxidative stress	Antioxidants (NAC, vitamin C, melatonin)	Reduced ROS injury	Mixed evidence	[6]
Inflammation	Cytokine/inflammatory inhibitors	Reduced tissue damage	Preclinical	[141]
Endothelial dysfunction	Barrier-protective agents	Vascular protection	Preclinical	[6]
Coagulopathy	Antithrombotic agents	Reduced thrombosis	Preclinical	[141]
Mitochondrial dysfunction	Mitophagy enhancers	Cellular recovery	Experimental	[154]
Tissue repair	Regenerative therapies	Enhanced healing	Preclinical	[155]

## Data Availability

No new data were created or analyzed in this study. Data sharing is not applicable to this article.

## Supplementary Materials

The following supporting information can be downloaded at: https://www.mdpi.com/article/10.3390/ijms27219804/s1.

## Abbreviations

The following abbreviations are used in this manuscript:

Abbreviation	Full term
ACD	Accidental Cell Death
AGER	Advanced Glycation End Product Receptor
AKI	Acute Kidney Injury
AOPP	Advanced Oxidation Protein Product
ARDS	Acute Respiratory Distress Syndrome
ATP	Adenosine Triphosphate
BAK	BCL-2 Antagonist/Killer
BAX	BCL-2-Associated X Protein
BCL-2	B-Cell Lymphoma 2
BID	BH3-Interacting Domain Death Agonist
cGAS	Cyclic GMP–AMP Synthase
CHOP	C/EBP Homologous Protein
CNS	Central Nervous System
CSR	Cellular Stress Response
DAMPs	Damage-Associated Molecular Patterns
DIC	Disseminated Intravascular Coagulation
DNA	Deoxyribonucleic Acid
ECM	Extracellular Matrix
ER	Endoplasmic Reticulum
GPX4	Glutathione Peroxidase 4
GSDMD	Gasdermin D
GSH	Glutathione
HIC	Heatstroke-Induced Coagulopathy
HMGB1	High-Mobility Group Box 1
HSEs	Heat shock Elements
HSF1	Heat shock Factor 1
HSPs	Heat shock Proteins
HSR	Heat shock Response
ICAM	Intercellular Adhesion Molecule
IL	Interleukin
LDH	Lactate Dehydrogenase
LPS	Lipopolysaccharide
MCP-1	Monocyte Chemoattractant Protein 1
MDA	Malondialdehyde
MLKL	Mixed Lineage Kinase Domain-Like Protein
MODS	Multiple Organ Dysfunction Syndrome
MOMP	Mitochondrial Outer Membrane Permeabilization
mtDNA	Mitochondrial DNA
NAC	N-Acetyl-L-Cysteine
NETs	Neutrophil Extracellular Traps
NF-κB	Nuclear Factor Kappa B
NLRP3	NLR Family Pyrin Domain Containing 3
NO	Nitric Oxide
NPs	Nanoparticles
PINK1	PTEN-Induced Kinase 1
PMVECs	Pulmonary Microvascular Endothelial Cells
PRRs	Pattern-Recognition Receptors
RAAS	Renin–Angiotensin–Aldosterone System
RAGE	Receptor for Advanced Glycation End Products
RCD	Regulated Cell Death
RIPK1	Receptor-Interacting Protein Kinase 1
RIPK3	Receptor-Interacting Protein Kinase 3
ROS	Reactive Oxygen Species
SERCA	Sarco/Endoplasmic Reticulum Ca^2+^-ATPase
SIRS	Systemic Inflammatory Response Syndrome
STING	Stimulator of Interferon Genes
TBSA	Total Body Surface Area
TF	Tissue Factor
TLRs	Toll-Like Receptors
TNF-α	Tumor Necrosis Factor Alpha
TRIF	TIR-Domain-Containing Adapter-Inducing Interferon-β
UPR	Unfolded Protein Response
VCAM	Vascular Cell Adhesion Molecule
vWF	von Willebrand Factor
ZBP1	Z-DNA-Binding Protein 1
