# Supplementary material for: From Proteostasis Collapse to Endothelial Failure: Mechanistic Pathways Linking Heat Stress, Regulated Cell Death, and Coagulopathy
Source: Int J Mol Sci. 2026 Oct 31;27(21):9804. doi: 10.3390/ijms27219804 (PMC13763785; doi:10.3390/ijms27219804)
Supplement: Supplementary file 1 [file ijms-27-09804-s001.zip › ijms-4660506-supplementary.pdf]

Literature Search Strategy

A systematic literature search of the PubMed, Scopus, and Web of Science databases was used to perform this narrative review. The English-language papers on heat stress, heatstroke, controlled cell death, endothelial dysfunction, and systemic inflammatory responses were the main focus of the literature search, which took place between January and May 2026. "Heatstroke," "hyperthermia," "proteostasis collapse," "regulated cell death," "apoptosis," "pyroptosis," "ferroptosis," "necroptosis," "PANoptosis," "DAMPs," "inflammasome," "oxidative stress," "endothelial dysfunction," "glycocalyx," "heatstroke-induced coagulopathy," and "multiple organ dysfunction syndrome" were towards the keyword combinations. Recent mechanistic research, experimental models, translational studies, and high-impact review publications related to heat-induced damage were prioritized. When deemed mechanistically relevant to extreme heat stress and systemic inflammatory injury, other papers from adjacent domains such as burn injury, sterile inflammation, and vascular injury were carefully included. Developing a hypothesis-driven integrative framework connecting proteostasis breakdown, controlled cell death, inflammatory amplification, endothelial dysfunction, and coagulopathy during severe heat stress was the goal of this review rather than offering a systematic review or meta-analysis.
